# Effectiveness of Self-guided Tailored Implementation Strategies in Integrating and Embedding Internet-Based Cognitive Behavioral Therapy in Routine Mental Health Care: Results of a Multicenter Stepped-Wedge Cluster Randomized Trial

**DOI:** 10.2196/41532

**Published:** 2023-02-03

**Authors:** Christiaan Vis, Josien Schuurmans, Bruno Aouizerate, Mette Atipei Craggs, Philip Batterham, Leah Bührmann, Alison Calear, Arlinda Cerga Pashoja, Helen Christensen, Els Dozeman, Claus Duedal Pedersen, David Daniel Ebert, Anne Etzelmueller, Naim Fanaj, Tracy L Finch, Denise Hanssen, Ulrich Hegerl, Adriaan Hoogendoorn, Kim Mathiasen, Carl May, Andia Meksi, Sevim Mustafa, Bridianne O'Dea, Caroline Oehler, Jordi Piera-Jiménez, Sebastian Potthoff, Gentiana Qirjako, Tim Rapley, Judith Rosmalen, Ylenia Sacco, Ludovic Samalin, Mette Maria Skjoth, Kristine Tarp, Ingrid Titzler, Erik Van der Eycken, Claire Rosalie van Genugten, Alexis Whitton, Enrico Zanalda, Jan H Smit, Heleen Riper

**Affiliations:** 1 Clinical, Neuro-, & Developmental Psychology Faculty of Behavioural and Movement Sciences Vrije Universiteit Amsterdam Amsterdam Netherlands; 2 Amsterdam Public Health research institute Amsterdam Netherlands; 3 Section for Research-based Innovation, Division of Psychiatry Haukeland University Hospital Bergen Norway; 4 GGZ InGeest Amsterdam Netherlands; 5 Regional Reference Center for the Management and Treatment of Anxiety and Depressive Disorders, FondaMental Advanced Centre of Expertise in Resistant Depression, Deparment of General and Academic Psychiatry Charles Perrens Hospital Bordeaux France; 6 Research Unit for Digital Psychiatry Deptartment of Clinical Research University of Southern Denmark Odense Denmark; 7 Centre for Mental Health Research The Australian National University Canberra Australia; 8 Department of Social Work, Education and Community Wellbeing Northumbria University Newcastle Upon Tyne United Kingdom; 9 London School of Hygiene and Tropical Medicine London United Kingdom; 10 Department of Medicine University of New South Wales Sydney Australia; 11 Sundhed.dk Odense Denmark; 12 Professorship Psychology & Digital Mental Health Technical University of Munich Munich Germany; 13 HelloBetter, GET.ON Institut für Online Gesundheitstrainings GmbH Berlin Germany; 14 Mental Health Center Prizren Prizren Kosovo; 15 Department of Nursing, Midwifery and Health Northumbria University Newcastle Upon Tyne United Kingdom; 16 Department of Psychiatry University Medical Center Groningen University of Groningen Groningen Netherlands; 17 German Depression Foundation Leipzig Germany; 18 Department of Psychiatry, Psychosomatics, and Psychotherapy Goethe Universität Frankfurt am Main Germany; 19 Psychiatry Amsterdam University Medical Center - location VUmc Amsterdam Netherlands; 20 Centre for Digital Psychiatry Mental Health Services of Southern Denmark Odense Denmark; 21 Faculty of Public Health and Policy London School of Hygiene and Tropical Medicine London United Kingdom; 22 Institute of Public Health Tirana Albania; 23 Black Dog Institute University of New South Wales Sydney Australia; 24 Catalan Health Service Barcelona Spain; 25 Digitalization for the Sustainability of the Healthcare System DS3-IDIBELL L’Hospitalet de Llobregat Barcelona Spain; 26 Faculty of Informatics, Multimedia and Telecommunications Universitat Oberta de Catalunya Barcelona Spain; 27 Department of Public Health Faculty of Medicine University of Medicine Tirana Albania; 28 Department of Internal Medicine University Medical Center Groningen University of Groningen Groningen Netherlands; 29 Department of Mental Health Local Health Authority Torino 3, ASLTO3 Torino Italy; 30 Department of psychiatry, Centre Hospitalier Universitaire de Clermont-Ferrand Expert center for bipolar disorder (Foundation FondaMental) University of Clermont Auvergne Clermont-Ferrand France; 31 Centre national de la recherche scientifique Clermont-Auvergne INP Institut Pascal (UMR 6602) Clermont-Ferrand France; 32 Department of Dermatology and Allergy Centre Odense University Hospital Odense Denmark; 33 Department of Clinical Psychology and Psychotherapy Friedrich-Alexander-Universität Erlangen-Nürnberg Erlangen Germany; 34 GAMIAN Europe Brussels Belgium

**Keywords:** common mental health disorders, internet-based cognitive behavioral therapy, iCBT, implementation strategies, tailored implementation, mobile phone

## Abstract

**Background:**

Internet-based cognitive behavioral therapy (iCBT) services for common mental health disorders have been found to be effective. There is a need for strategies that improve implementation in routine practice. One-size-fits-all strategies are likely to be ineffective. Tailored implementation is considered as a promising approach. The self-guided integrated theory-based Framework for intervention tailoring strategies toolkit (ItFits-toolkit) supports local implementers in developing tailored implementation strategies. Tailoring involves identifying local barriers; matching selected barriers to implementation strategies; developing an actionable work plan; and applying, monitoring, and adapting where necessary.

**Objective:**

This study aimed to compare the effectiveness of the ItFits-toolkit with implementation-as-usual (IAU) in implementing iCBT services in 12 routine mental health care organizations in 9 countries in Europe and Australia.

**Methods:**

A stepped-wedge cluster randomized trial design with repeated measures was applied. The trial period lasted 30 months. The primary outcome was the normalization of iCBT delivery by service providers (therapists, referrers, IT developers, and administrators), which was measured with the Normalization Measure Development as a proxy for implementation success. A 3-level linear mixed-effects modeling was applied to estimate the effects. iCBT service uptake (referral and treatment completion rates) and implementation effort (hours) were used as secondary outcomes. The perceived satisfaction (Client Satisfaction Questionnaire), usability (System Usability Scale), and impact of the ItFits-toolkit by implementers were used to assess the acceptability of the ItFits-toolkit.

**Results:**

In total, 456 mental health service providers were included in this study. Compared with IAU, the ItFits-toolkit had a small positive statistically significant effect on normalization levels in service providers (mean 0.09, SD 0.04; *P*=.02; Cohen *d*=0.12). The uptake of iCBT by patients was similar to that of IAU. Implementers did not spend more time on implementation work when using the ItFits-toolkit and generally regarded the ItFits-toolkit as usable and were satisfied with it.

**Conclusions:**

The ItFits-toolkit performed better than the usual implementation activities in implementing iCBT services in routine practice. There is practical utility in the ItFits-toolkit for supporting implementers in developing and applying effective tailored implementation strategies. However, the effect on normalization levels among mental health service providers was small. These findings warrant modesty regarding the effectiveness of self-guided tailored implementation of iCBT services in routine practice.

**Trial Registration:**

ClinicalTrials.gov NCT03652883; https://clinicaltrials.gov/ct2/show/NCT03652883

**International Registered Report Identifier (IRRID):**

RR2-10.1186/s13063-020-04686-4

## Introduction

### Background

Common mental health disorders such as depressive disorder and anxiety account for a large proportion of the global burden of disease [1-3]. Effective evidence-based treatments exist, but access to care has become a critical issue for countries across Europe and the world. In the last 2 decades, effective clinical innovations that may help overcome this challenge have been developed at high rates [4]. Internet-based cognitive behavioral therapy (iCBT) for common mental disorders has a wide evidence base that can potentially increase the reach and accessibility of mental health services with clinical effects comparable with face-to-face psychotherapy [5-8]. Despite the evidence base, and although examples of successful implementation exist, widespread use of iCBT services in routine mental health care lags behind expectations [9,10].

When an organization decides to adopt iCBT treatments, implementation strategies are often focused on the technical infrastructure and educational training of service providers. Service providers commonly receive technical training that focuses on how to use the web-based iCBT platform. Although important, these strategies may not necessarily address the most urgent barriers to implementation. Often, it requires learning new communication skills [11] and reconfiguring existing organizational procedures and clinical operating guidelines. Successful implementation of iCBT platforms, therefore, requires an integrative approach that considers a wide range of barriers.

In general, iCBT services are not implemented and delivered in isolation. They impact and interact with various aspects of the health care service delivery system. Implementing iCBT services in routine mental health care practice is a complex process that affects multiple actors, such as service providers, clinical directors, policy makers, insurers, managers, administrators, and patients, and does so at multiple levels [12,13]. In the complex changes required to deliver the new service, many different factors affect iCBT implementation. The scientific literature on implementation barriers is relatively rich and identifies factors such as available resources; attitudes and capabilities of service referrers [14,15] and mental health service providers [11,16,17]; and other barriers that exist at the system, organizational, service provider, and patient levels [18]. These factors are also likely to change over time. Despite this rich scientific literature, we have an incomplete understanding of how these factors interact with iCBT service delivery and how effective different implementation strategies are in targeting implementation barriers.

Implementation, seen as a process by which people bring new or modified practices into operation [19], takes place in a dynamic context and is susceptible to barriers that vary from setting to setting and over time [20-24]. Tailored implementation is a process by which implementation work takes into account factors in the local context in which the new service is to be integrated and embedded. Examples of such factors include financial and time constraints, needs and capabilities of team members, specific organizational procedures, structures and habits, and values and beliefs of certain stakeholder groups. Innovations can be implemented more quickly and efficiently by systematically addressing the factors that are most likely to impede and facilitate the uptake in the context of the local setting [25,26]. Evidence of the effectiveness of tailored implementation came from a Cochrane review (n=17 studies) of tailored implementation strategies that focused on implementing clinical guidelines in various clinical settings [27]. Using health professionals’ adherence to guidelines as an indicator of implementation success, the review found a pooled odds ratio of 1.56 (95% CI 1.27-1.93; *P*<.001) showing that tailored implementation leads to better implementation outcomes compared with doing nothing or applying implementation strategies that are not tailored to local determinants. The authors concluded that on face value, tailored implementation “can be effective, but the effect is variable and tends to be small to moderate” [27]*.*

To date, mostly expert-driven models of tailored implementation have been developed and studied. In models that involve expert-driven tailoring, experienced implementation researchers or implementation practitioners play a prominent role in guiding the tailoring process, designing the implementation strategy, and applying the strategy. Generally, these experts are external to the organization, not involved in the development of the intervention or guideline that is to be implemented, and they do not necessarily have in-depth knowledge of or experience with the specific context such as mental health care. Their primary field of expertise lies in facilitating, advising, or evaluating the implementation of complex interventions in various medical fields including primary and specialized health care. An example of expert-driven tailoring was applied in the Tailored Implementation for Chronic Diseases project [28]. In this project, a team of experienced implementation researchers facilitated the identification and matching of barriers to implementing strategies. It was concluded that the implementation activities resulted in “improvements on some outcomes, but they had overall little observable impact on primary or secondary [patient-level] outcomes” [28]. Studies of expert-driven tailored implementation have also been conducted in mental health settings. For example, Sinnema et al [29] found that an implementation program tailored to address barriers perceived by general practitioners (GPs) can improve recognition of anxiety and depression in patients presenting for treatment in primary care (odds ratio 1.60, 95% CI 1.01-2.53). In this study, barriers were identified on the basis of a literature review by the research team and by trained interviewers who interviewed GPs. The tailored implementation intervention consisted of peer group supervision as well as periodic telephone consultations facilitated by the research team and experienced GPs that were used to iteratively discuss the identified barriers and suggest possible solutions to address them [30].

Given this evidence base, there were limitations to the expert-driven model of tailored implementation. For example, external experts may be less adept at identifying context-specific barriers than local implementers, possibly leading to less effective implementation strategies. In addition, external experts may not know or have access to relevant local stakeholders, or they may be regarded as outsiders, possibly limiting the acceptability of specific implementation strategies by local stakeholders. Furthermore, expert-driven models of tailoring might have scalability issues and practical constraints because of the limited availability of experts to coordinate and facilitate tailoring of implementation strategies. Alternatively, tailoring more intensively to specific settings can be an alternative to improving the effectiveness of tailored implementation, but it is likely to be very costly [28].

To overcome these limitations and to potentially improve the effectiveness of tailored implementation strategies, the integrated theory-based Framework for intervention tailoring strategies toolkit (ItFits-toolkit) was developed. As part of the Horizon 2020 ImpleMentAll (IMA) project [31], the ItFits-toolkit was specifically designed as a web-based self-guided tool for local implementers. The ItFits-toolkit provided a systematic and flexible approach embedded in theoretical and conceptual ideas from the field of implementation science, including Normalization Process Theory (NPT) [32]. The toolkit does not require prior experience in, or knowledge of, implementing new clinical interventions in routine care, and it supports local implementers in developing evidence-informed implementation strategies that are tailored to local needs.

### Objective

In this study, we examined the effectiveness of the ItFits-toolkit and answered the following research question: does the use of the ItFits-toolkit lead to better outcomes than implementation-as-usual (IAU) in implementing iCBT services in routine mental health care? Implementation effectiveness was defined as the extent to which the iCBT services were regarded as being a normal part of mental health care practice by service providers. We hypothesized that ItFits-toolkit use would be associated with increased normalization of iCBT services into practice. Parallel to this effectiveness study, an in-depth qualitative process evaluation was conducted, focusing on engagement, embedding, and integration of the ItFits-toolkit by implementers [33,34].

## Methods

A multicenter trial was conducted in Albania, Australia, Denmark, France, Germany, Italy, Kosovo, the Netherlands, and Spain. This study was conducted between March 2018 and March 2021. The study protocol is published elsewhere [31].

### Study Design

**Figure 1:**
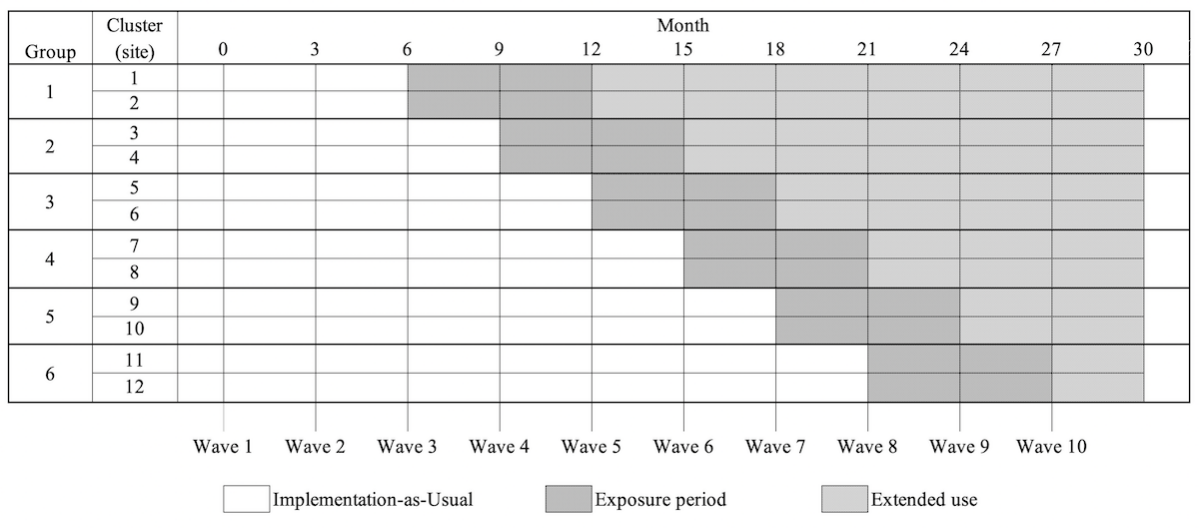
Stepped-wedge cluster randomized controlled trial design.

A stepped-wedge cluster randomized controlled trial design was applied [35]. The main design principles are shown in Figure 1. Over a period of 30 months, the ItFits-toolkit was sequentially rolled out into 6 groups of 12 organizations (clusters). The clusters were randomly allocated with an interval of 3 months, at which the clusters crossed over from the control condition (IAU) to the experimental condition (ItFits-toolkit). Data were collected in 10 waves with a 3-month interval period (waves 1-10) to strike a balance between measuring change over time and the measurement burden imposed on the study participants.

### Settings

A total of 12 mental health service delivery organizations in 9 countries were included in the study (Table 1). All organizations embarked on implementing an iCBT-based prevention or treatment service for common mental disorders. Of the 12 organizations, 1 (8%) was unable to participate because its iCBT platform was technically not ready for implementation when the first data collection wave commenced. This organization was replaced by a backup mental health service delivery organization available within the IMA consortium that was ready to implement its iCBT service. The iCBT services that were implemented targeted people with mild to severe depressive disorders, anxiety disorders, substance abuse, or somatic symptom disorders. One service included a prevention approach that addressed symptoms for developing mental health disorders. All iCBT services were based on cognitive behavioral therapy, covering 4 main working mechanisms: psychoeducation, techniques invoking behavioral change, a cognitive component, and relapse prevention [36]. All services were internet-based, using web-based delivery platforms, smartphone-based apps, or a combination of both technologies. Various guidance modalities were embedded in the iCBT services, ranging from unguided with minimal technological and administrative support to therapist-guided and blended treatments, where web-based modules and face-to-face therapy were integrated into one treatment protocol [37,38]. Patient pathways, diagnostic criteria, meaningful therapeutic exposure, and stopping rules followed local treatment manuals and clinical guidelines. The specific operationalization differed per service in response to local requirements and preferences (refer to the published study protocol [31] for more information).

**Table 1:** Overview of mental health organizations and main iCBT^a^ characteristics.

Organization	Country codes	Prevention	Primary care	Secondary care	iCBT platform or program	Unguided	Guided	Blended
IMA0101	AL^b^	—^c^	—	✓^d^	iFight Depression	—	✓	—
IMA0201	AU^e^	✓	✓	—	FitMindKit	✓	—	—
IMA0301	DE^f^	—	✓	—	iFight Depression	—	✓	—
IMA0302	DE	✓	—	—	Get.On or HelloBetter	—	✓	—
IMA0401	DK^g^	—	—	✓	NoDep & Fearfighter or MindDistrict	—	✓	—
IMA0501	ES^h^	—	—	✓	Super@tuDepresión	—	✓	—
IMA0502	ES	—	✓	—	Super@tuDepresión	—	✓	—
IMA0601	FR^i^	—	—	✓	MoodBuster	—	✓	✓
IMA0701	IT^j^	—	—	—	iFight Depression	—	✓	—
IMA0801	NL^k^	—	✓	✓	MindWay using MindDistrict	—	—	✓
IMA0802	NL	—	✓	—	MySelf or Master your symptoms	—	✓	—
IMA0901	XK^l^	✓	—	✓	iFight Depression	—	✓	—

^a^iCBT: internet-based cognitive behavioral therapy.

^b^AL: Albania.

^c^—: Not applicable.

^d^✓: applicable.

^e^AU: Australia.

^f^DE: Germany.

^g^DK: Denmark.

^h^ES: Spain.

^i^FR: France.

^j^IT: Italy.

^k^NL: the Netherlands.

^l^XK: Kosovo.

### Study Participants

Two types of participants were included in the study: (1) implementers, that is, local staff who facilitated the implementation of the iCBT service, and (2) mental health service providers such as therapists who were involved in iCBT service delivery.

For each of the 12 organizations, a team of up to 5 staff members was appointed as implementers. One team member was appointed as the implementation lead and coordinated the work. Implementers were directly involved in the development, coordination, and execution of local implementation activities such as designing and distributing iCBT information leaflets or developing training materials for referrers and therapists. Implementers were not required to have prior experience in, or specialist knowledge of, implementing iCBT services, but were expected to have working knowledge of the service they were implementing. Implementers could have different functions or roles in the organization, that is, manager, researcher, or clinician. Implementers were expected to have a proficient command of the English language to be able to use the ItFits-toolkit.

Mental health service providers were eligible to be included if they had a distinct role in delivering iCBT to patients, including clinicians, psychologists, GPs, psychiatrists, psychiatric nurses, staff in a supporting role (eg, administrators), or IT professionals involved in the operation of the technical aspects of the iCBT services.

Local research teams recruited both implementers and service providers with the support of a central research team overseeing the trial. To avoid contamination between the implementation team and the target population of the toolkit, participants could not act as both implementers and service providers.

### Intervention: ItFits-Toolkit

**Figure 2:**
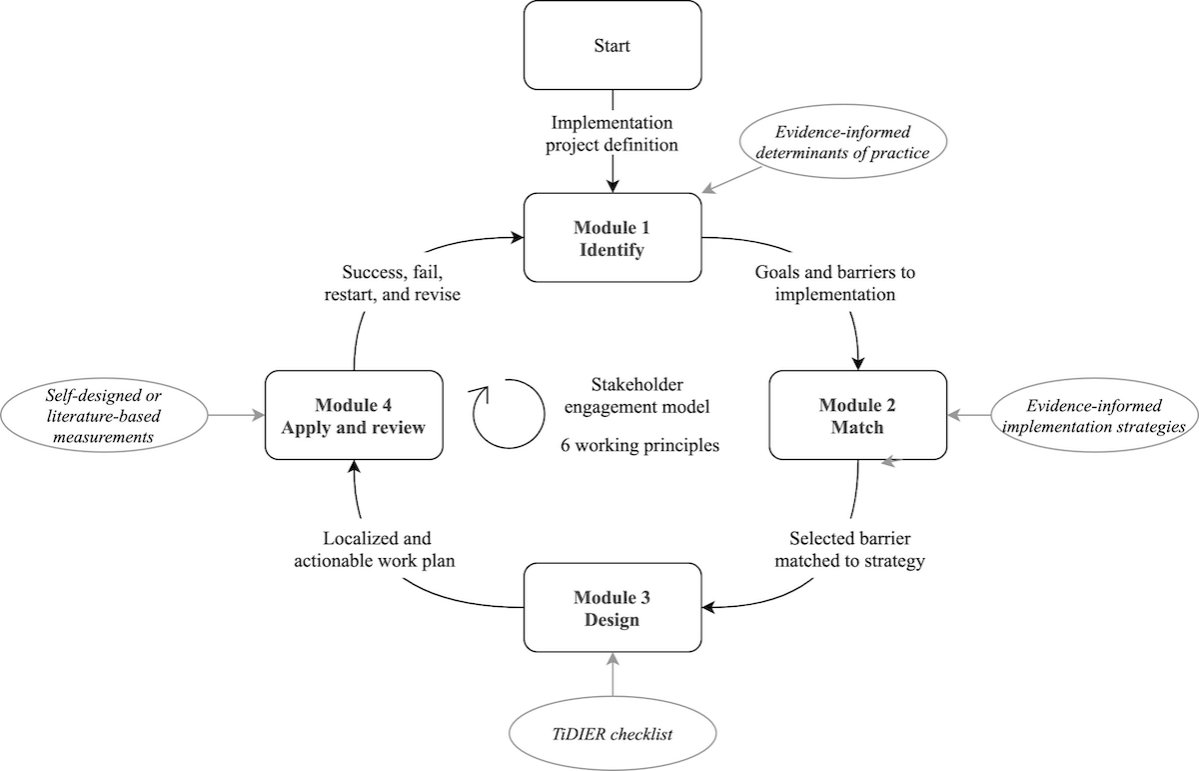
Integrated Theory-based Framework for Implementation Tailoring Strategies toolkit (ItFits-toolkit) process flow and main working mechanisms. TiDIER: Template for Intervention Description and Replication.

The ItFits-toolkit consists of four web-based modules that guide users through the tailoring process: (1) identifying and prioritizing implementation goals and barriers to achieving these goals, (2) matching barriers to implementation strategies, (3) designing a work plan to carry out the strategies, and (4) applying strategies and reviewing progress. An overview of the main components of the ItFits-toolkit is summarized in Figure 2 and Textbox 1. Within the respective modules, implementers work with literature-based materials, including a repository of barriers [18] and implementation strategies [39-41]. The work plan developed in module 3 was structured using the Template for Intervention Description and Replication (TIDieR) checklist [42]. Within the 4 modules, ideas from NPT [32] are integrated, including stakeholder involvement. In each module, implementers work through a 3-step iterative stakeholder consultation process using several methods (eg, brainstorming, structured group discussions, or surveying) to reach the best possible outcome. A web-based surveying tool is integrated in the ItFits-toolkit to collect views from stakeholders throughout each module and to collect information on indicators to assess the effects of the tailored implementation strategies in module 4. Notes, audio recordings, and other relevant materials can be uploaded to document the decisions and progress made in each module. In developing the toolkit, a balance was sought between theoretical foundation, practical orientation, and usability. The ItFits-toolkit was built from scratch before the start of recruitment to the trial and underwent various rounds of conceptual and technical piloting with various user groups representing the perspectives of implementers, clinical stakeholders, and researchers. More information regarding the toolkit is available on the project website [43] and in the study protocol [31]. This toolkit is freely accessible [44].

**Core working components of the integrated theory-based Framework for intervention tailoring strategies toolkit (ItFits-toolkit).**

Core working components of the ItFits-toolkit:

- Nonstandardized, systematically guided step-by-step process
- Stakeholder-based cocreation
- Tools to identify local barriers, consult stakeholders, and match to suitable strategies
- Evidence-informed materials on barriers, strategies, and intervention planning
- Six working principles: pragmatic, flexible, focused, openness, organized, and different

### Control Condition: IAU

The IAU functioned as the control condition to test the effectiveness of the ItFits-toolkit. The IAU referred to any existing activities that the organizations were engaged in to implement the iCBT services in routine care. During the trial, IAU was mostly concerned with communication and dissemination activities, training, education, and further adapting the services to the local requirements.

### Exposure

At crossover (Figure 1), the implementers received access to the toolkit following an introductory training. The training covered the ItFits-toolkit working principles and technical instructions to get started. A period of 6 months was chosen as the exposure period to balance the practical and financial feasibility of the study, with realistic opportunities for implementers to gain experience with the toolkit and being exposed to the core components of the toolkit. Adequate exposure to the ItFits-toolkit was defined as the implementers completing modules 1, 2, and 3 within the exposure period (Figure 2). During the exposure period, the sites received technical support in the form of monthly conference calls. As with the introductory training, the calls were limited to the technical use aspects of the toolkit and did not address any specific implementation advice such as which barriers to address or which strategy to use. The introductory training and calls were provided by members of the central research team involved in the development of the ItFits-toolkit.

Data from the web-based platform showed that out of the 12 implementation teams, 10 (83%) progressed from at least one of their projects to module 4 during the exposure period. Qualitative data revealed that 2 of the remaining teams were in the process of applying their strategies, without recording it on the platform. All teams continued to use the toolkit after they completed the exposure period. A total of 31 projects (range 1-6 per organization) were initiated using the ItFits-toolkit. The term “project” referred to a process initiated in the ItFits-toolkit to develop and apply a tailored strategy in relation to the iCBT service. For 12 projects, a full cycle of all 4 ItFits-toolkit modules was completed. A further 8 projects were partially completed (up to and including module 3, at which stage the designed strategy was being applied and monitored).

### Outcome Measures

The primary outcome was the degree of normalization among service providers. Normalization, as conceptualized by NPT, concerned the actions that people engage in to integrate and embed new practices in their work so that these new practices become a normal part of their daily workflow [32]. This outcome indicator was chosen because of the prominent role that service providers play in providing iCBT services, with the expectation that they would adapt their way of working to accommodate the delivery of iCBT. The outcome of normalization is to be understood as the degree to which service providers perceive the delivery of iCBT as a normal, integrated, well-supported, and sustainable part of their work routine. Normalization was measured using the Normalization Measure Development (NoMAD) questionnaire [45,46]. The NoMAD is a brief self-reported questionnaire with 20 items addressing the 4 generative mechanisms involved in implementation processes, as conceptualized by NPT: coherence, cognitive participation, collective action, and reflexive monitoring. Items were rated on a 5-point Likert scale (1=completely agree to 5=completely disagree, with 3=neutral). The NoMAD has high internal consistency in various health care settings and languages [46,47], including in mental health [48].

### Secondary Outcomes

To complement the primary outcome, we assessed the effectiveness of the ItFits-toolkit using measures of uptake of the iCBT service by patients (referral and completed treatments with adequate exposure levels) and implementation efficiency (operationalized by hours spent by implementers). These were used as secondary outcomes.

The satisfaction, usability, and impact of the ItFits-toolkit, as perceived by the implementers, were assessed to explore the extent to which the toolkit could fulfill implementers’ needs and expectations in developing tailored implementation strategies. Satisfaction was measured using the short version of the Client Satisfaction Questionnaire (CSQ-3 items) [49-52]. The CSQ has good psychometric properties and has been tested in numerous studies and diverse samples [51,53,54]. Usability was measured using the System Usability Scale (SUS; [55,56]) to determine the degree to which the toolkit was perceived as usable. Perceived impact was assessed using a Visual Analog Scale (VAS) to explore whether the implementation strategies developed using the ItFits-toolkit had an impact and were helpful from the perspective of the implementers. Further details of the outcomes and measurement properties of each instrument are provided in Table 2.

**Table 2:** Overview of the primary, secondary, and exploratory outcomes.

Outcome			Staff		Organization		Instrument		Baseline		Repeated measures		End of exposure
**Primary outcome**													
	Degree of normalization	✓^a^		—^b^		NoMAD^c^		✓		✓		—	
**Secondary outcomes**													
	Uptake (referral and completion)	—		✓		iCBT^d^ platform		✓		✓		—	
	Implementation costs	—		✓		Questionnaire effort and costs		✓		✓		—	
**Exploratory outcomes**													
	Exposure to ItFits-toolkit^e^	—		✓		Event-based platform log files		Continuous		—		—	
	Usability	—		✓		SUS^f^ (10 items)		—		—		✓	
	Satisfaction	—		✓		CSQ^g^ (3 items)		—		—		✓	
	Perceived impact and helpfulness of ItFits-toolkit	—		✓		VAS^h^ statements		—		—		✓	

^a^✓: applicable.

^b^—: Not applicable.

^c^NoMAD: Normalization Measure Development. Native language versions were developed using a standardized forward and backward translation protocol.

^d^iCBT: internet-based cognitive behavioral therapy.

^e^ItFits-toolkit: integrated theory-based Framework for intervention tailoring strategies toolkit.

^f^SUS: System Usability Scale.

^g^CSQ: Client Satisfaction Scale.

^h^VAS: Visual Analog Scale.

### Sample Size Considerations

This study had a fixed number of 12 clusters by design. Mental health service delivery organizations participated based on their commitment to implementing iCBT services. For service providers, the sample size needed for sufficient power to test the use of the ItFits-toolkit on the degree of normalization (ie, NoMAD) was obtained from a power analysis using simulated data. As there was no prior knowledge concerning the NoMAD in detecting changes in normalization, we assumed a 5% increase in absolute normalization scores and an increase in the 3-month growth rate from 0.05 to 0.10. A cluster sample size of 15 service providers for each of the 12 mental health organizations per wave was estimated to be sufficient to achieve 80% power to detect the effect using a 2-sided test with a significance level α of .05. The first 2 data collection waves were used to obtain a stable sample, and recruitment was closed in wave 3. Replacements were sought for those service providers who had dropped out of the study.

### Data Management

Nested within the service delivery organizations, data were collected from implementers and service providers. Some data and outcomes (demographics, satisfaction, and usability) were collected once, whereas data on the primary and secondary outcomes were assessed every 3 months (Table 2). All questionnaires were translated using a forward-backward translation procedure [31]. All data were collected through a secure web-based central Data Collection System (DCS), which allowed a standardized and structured data collection process. The DCS was developed specifically for this study and designed to prevent missing values or false entries and to enable automatic anonymization.

Deidentified data supporting the findings of this study are available upon request. Investigators interested in using the data must provide a methodologically sound proposal directed to the corresponding author. It is necessary to sign a data use or sharing agreement. The documentation on the ItFits-toolkit as well as all translations of the NoMAD are available for public use and can be accessed on the project’s website [57]. The ItFits-toolkit is freely available [44].

### Data Analysis

Data for the primary outcome were analyzed using a 3-level linear mixed-effects modeling (LMM) approach [35] with normalization as the dependent variable and time (as a discrete variable) and intervention (ie, the ItFits-toolkit use) as independent variables. To account for expected intervention lag effect, a fractional term for the ItFits-toolkit use parameter was used to reflect the 6-month exposure time (0, 0.5, and 1). To account for a correlation structure in the outcome involving 3 nested levels, repeated measurements (L1) were clustered at the level of service providers (L2), and service providers were clustered at the organization level (L3). A temporal effect was assessed by testing the null hypothesis that the normalization level was constant over time when controlling for the effect of the ItFits-toolkit using ANOVA. A 2-sided test with a significance level α of .05 was used. Cohen *d* was used as a measure of the effect by which the modeled estimate was standardized by the pooled within-organization SD of the NoMAD scale at wave 1. Standard cutoff levels were applied (small effect: Cohen *d*≤0.2, medium effect: 0.2<Cohen *d*<0.8, and a large effect: Cohen *d*≥0.8). Before analyzing and opening the data, various potentially confounding moderators were conceptually explored by the central research team following 2 workshops, using preliminary information from the qualitative process evaluation [33]. The role of staff in service delivery was selected as a potential moderator. Specifically, we assumed that staff who were more directly involved in iCBT service delivery such as psychologists and psychiatrists were likely to undergo a more extensive change process to normalize iCBT service delivery than those at a larger distance to the service delivery process such as referrers and administrators.

For the secondary outcomes, service uptake (iCBT referral and completion) and effort (hours) were modeled following the same approach as for the primary outcome, except that a 2-level LMM was applied as these measures were collected at the organization level only (ie, not at the level of staff members but only waves [Level 1] clustered at the organization level [Level 2]). For exploratory purposes, measures of exposure to the ItFits-toolkit (module-based log as an indication of use), CSQ, SUS, and perceived impact and helpfulness of the ItFits-toolkit were assessed descriptively as an indication of usability, satisfaction, impact, and helpfulness of the ItFits-toolkit from the perspective of implementers. Perceived impact and helpfulness were measured using a VAS, with a continuous scale ranging from 1.0 10.0. The scale scores for SUS and CSQ were calculated using the respective prescribed scoring systems. For CSQ, summed item rating scores were used [56]. For SUS, the summed item ratings were converted to a 0 to 100 scale using a curved grading scale, with 68 points to be interpreted as neutral [58].

All observed data were included in the analyses following the intention-to-treat principle. We relied on the capability of linear mixed-effects models to estimate model parameters in case of missing values under the Missing at Random assumption [59]. Data cleaning and analyses were performed using R (version 4.0.2; R Foundation for Statistical Computing) [60] in RStudio [61] using the following packages: *dplyr* [62], *psych* [63], *ggplot2* [64], and *lmerTest* [65].

### Ethics Approval and Consent to Participate

Each local research team translated and adapted the generic study protocol to the local requirements and submitted it for review to competent local medical ethical review committees. All committees either approved the study or provided a confirmation letter that it was exempt from approval as the study was not considered as medical research. A portfolio of the ethical documentation can be accessed through Deliverable 23 (D9.1, H – Requirement No. 1), which was submitted to the European Commission (Horizon 2020, Grant Agreement 733025). The study participants signed an informed consent form indicating the purpose of the study and the nature, use, and management of their data. The study protocol was published [31], and the study was registered with ClinicalTrials.gov (NCT03652883).

## Results

### Recruitment and Sample Characteristics

A total of 39 implementers in 12 mental health service delivery organizations used the ItFits-toolkit to implement the iCBT services. The group had a mean age of 42.6 (SD 10.1) years, and 69% (27/39) of the implementers were female. More than half (23/39, 59%) of the implementers had ≥6 years of work experience in mental health and were appointed as general project managers (13/39, 33%) or clinical researchers (12/39, 31%).

A total of 456 iCBT service providers were included in this study (Table 3). The response rate of the service providers was high (78% across all 10 waves), resulting in 2884 complete data points. Approximately 30.9% (141/456) of the service providers were replaced because of study dropout during waves 3 to 10. The group had a mean age of 41 (SD 11) years, and 68.6% (313/456) of the service providers were female. Most service providers were therapists (257/456, 56.3%). A total of 73.9% (337/456) had no experience with iCBT delivery before the study.

**Table 3:** Demographics of service providers included in the study (N=456).

Variable			Values
**Sex, n (%)**			
	Female	313 (68.6)	
	Male	143 (31.3)	
Age (years), mean (SD; range)			41.26 (11.08; 18-72)
**Work experience (years), n (%)**			
	<1	56 (12.2)	
	1-2	79 (17.3)	
	3-5	84 (18.4)	
	6-10	75 (16.4)	
	11-15	75 (16.4)	
	>15	87 (19)	
No Prior iCBT^a^ experience, n (%)			337 (73.9)
**Role in iCBT delivery, n (%)**			
	Therapist, etc	257 (56.3)	
	Referrer	159 (34.8)	
	Administrator	36 (7.8)	
	ICT^b^	4 (0.01)	

^a^iCBT: internet-based cognitive behavioral therapy.

^b^ICT: information and communication technology.

### Primary Outcome: Normalization

**Figure 3:**
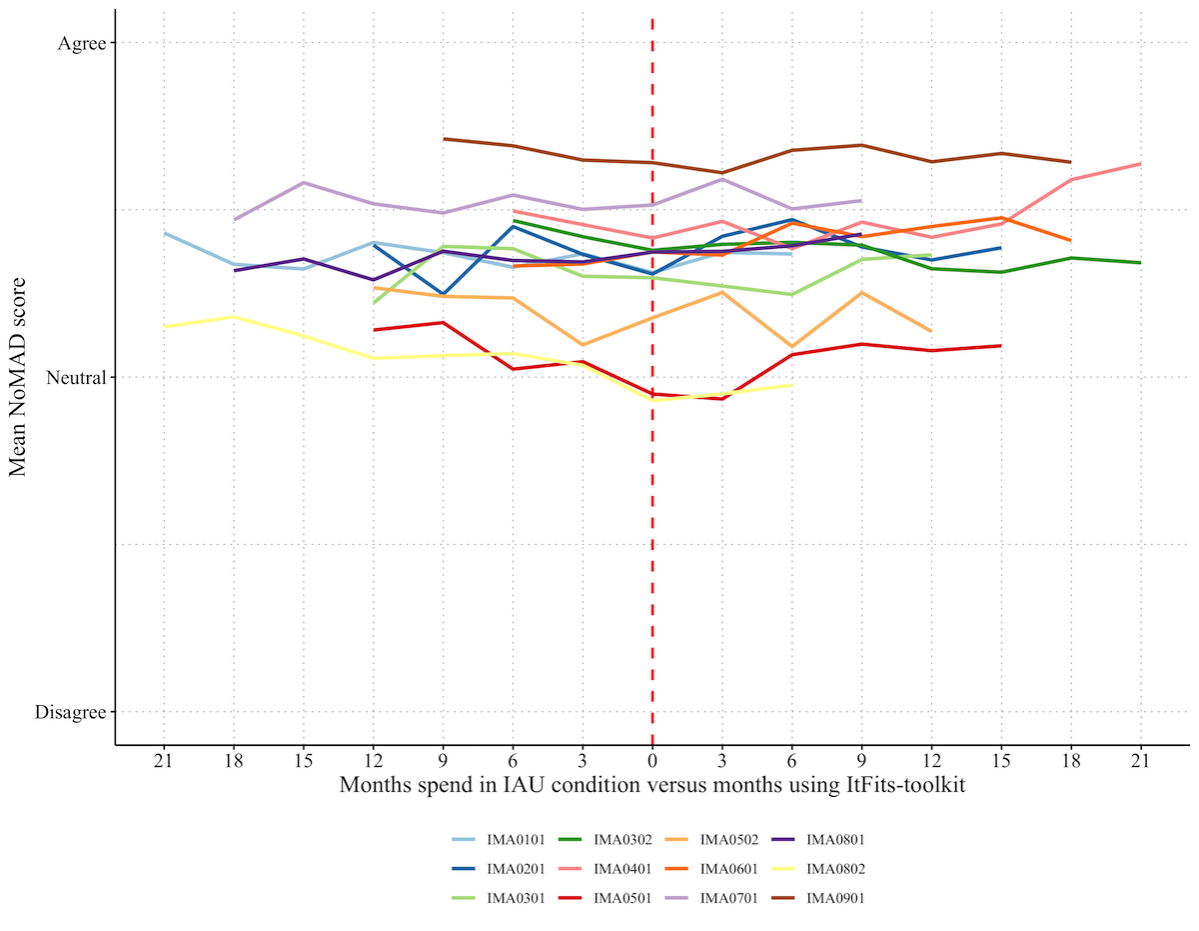
Mean Normalization Measure Development (NoMAD) score per mental health service delivery organization across time. IAU: implementation-as-usual; ItFits-toolkit: Integrated Theory-based Framework for Implementation Tailoring Strategies toolkit.

On average, service providers (n=456) scored normalization of the iCBT service slightly above neutral both during IAU (mean_IAU_ 3.63, SD 0.72; n*_observations_*=1242) and when the ItFits-toolkit was used (mean_ItFits_ 3.67, SD 0.76; n*_observations_*=1642). Figure 3 shows that mean normalization levels were relatively stable over time, both during the IAU and when using the ItFits-toolkit. The figure also shows considerable differences between organizations, with average normalization scores ranging from 3.11 to 4.32 on a Likert scale of 1 to 5. The item and scale scores are included in Multimedia Appendix 1 [35].

Using the LMM, we found that overall and at the end of the study period, the ItFits-toolkit had a small positive statistically significant effect on normalization levels (mean_ItFits_ 0.09, SD 0.04; *t*_2514_=2.34, 2-tailed; *P*=.02; Cohen *d*=0.12) when compared with IAU. The model definitions and outcomes have been included in Multimedia Appendix 1. When testing the levels of normalization over time and controlling for the ItFits-intervention effect using ANOVA, a significant temporal effect was apparent (*χ*^2^_9_=25.7; *P*=.002). Over time, the levels of normalization decreased slightly in the IAU condition (mean_IAU_ −0.13, SD 0.06; *t*_2454_=−2.27; *P*=.02). Tailored implementation, as operationalized in the ItFits-toolkit, partially canceled out this negative trend over time. Subgroup analysis showed that the ItFits-toolkit had no statistically significant effect on normalization in service providers that were directly involved in iCBT delivery (ie, therapists; mean_group 1_ −0.02, SD 0.07; *t*_1364_=1.52; *P*=.81) or service providers that were more remote from the delivery process (ie, referrers, IT personnel, administrators, etc; mean_group 2_ 0.10, SD 0.06; *t*_1141_=2.02; *P*=.06).

### Secondary Outcomes

#### Uptake

During the IAU condition, 3256 patients were referred to the iCBT services, of whom 588 (18.05%) received adequate exposure to the iCBT services. During the ItFits-toolkit condition, 3935 individuals were referred to the iCBT services, of whom 842 (21.39%) received adequate exposure. Over the course of the trial period, 7191 patients were referred and received log-in credentials to the iCBT services. Following local treatment protocols, 19.88% (1430/7191) patients received meaningful exposure to the iCBT services, 72.59% (5220/7191) dropped out of the iCBT service, 5.71% (410/7191) were using the iCBT service, and 4.35% (313/7191) did not start with the iCBT service when data collection was closed. Upon inspection of the data, changes in uptake over time followed an inconsistent and variable pattern, and no clear effect was visible with regard to the introduction of the ItFits-toolkit. This was confirmed in our modeling, which showed that differences in referral (mean_Referral_ 21.50, SD 26.71; *t*_102.9_=0.81; *P*=.42) or completion rates (mean_Completion_ 10.87, SD 5.83; *t*_105.8_=1.87; *P*=.07) between IAU and ItFits-toolkit use were not statistically significant. For both outcomes, no temporal effect in uptake was observed (*χ*^2^_9Referral_=8.3, *P*=.51, and *χ*^2^_9Completion_=8.3, *P*=.50). The cumulative uptake levels and model specifications are included in Multimedia Appendix 2 [35].

#### Effort

As a proxy for assessing the efficiency of the ItFits-toolkit compared with IAU, the hours implementation teams spent on implementation were recorded. Over the entire study period, 20,277.5 hours were spent on implementation activities. With an assumed average of 1650 hours for a yearly full-time equivalent (FTE) position, the pooled effort corresponded to 4.9 FTE (per year; Multimedia Appendix 3 [35]) spent by the core implementation teams in total, ranging from 0.05 FTE to 2.46 FTE across the mental health service delivery organizations. Similar to uptake, the time spent on implementation activities followed an inconsistent pattern, and there was no clear effect of the ItFits-toolkit. This was confirmed in our modeling, which showed that the differences in hours spent on implementation between IAU and ItFits-toolkit use were not statistically significant (mean_difference in effort_ 45.88, SD 41.62; *t*_102.9_=1.10; *P*=.27). Details and model specifications are included in Multimedia Appendix 3.

### Perceived Usability, Satisfaction, Impact, and Helpfulness of the ItFits-Toolkit

Implementers found the toolkit generally usable (mean_SUS-10_ 77.3 out of 100, SD 14.2; cutoff=68) and were satisfied with it (mean_CSQ-3_ 7.4 out of 12, SD 0.9). Implementers graded the impact of the ItFits-toolkit in supporting them in achieving their implementation objectives and addressing barriers on average a 6.5 (SD 1.8; 10-point VAS), with a slightly higher average rating for its support in addressing barriers (mean 6.9, SD 1.3) versus objectives (mean 6.1, SD 2.5; 10-point VAS). Implementers regarded the toolkit in general as helpful, rating it 7.1 (SD 0.8; 10-point VAS) on average. The use and perceived added value of the ItFits-toolkit in implementation of iCBT services was the central focus of the process evaluation [33].

## Discussion

### Principal Findings

This study sought to examine whether the ItFits-toolkit leads to better implementation outcomes than IAU in implementing iCBT services. In comparison with IAU, the ItFits-toolkit has a small statistically significant effect on normalization levels in iCBT service providers. The toolkit did not have an effect on iCBT service uptake by patients, and the implementers did not spend more time using the toolkit. ItFits-toolkit users regarded the toolkit generally as usable and were satisfied with it. These findings fit the general pattern across tailoring studies [27].

To the best of our knowledge, this was the first study to investigate the effectiveness of self-guided tailored implementation of eMental health services supported by a web-based implementation toolkit using a standardized, validated, and quantitative primary implementation outcome in service delivery staff in mental health settings. Practically, these findings can contribute to implementing iCBT services into routine care by delivering a functional and technically stable toolkit. The toolkit was easy to use, provided implementers with flexible ways to structure and infuse their work with scientific knowledge, and involved relevant stakeholders in developing and executing tailored implementation strategies. Findings indicated that implementation can be improved with a self-guided toolkit that enhanced implementation outcomes without extra investments of effort. Despite the small effect and the need for further research to better understand and optimize outcomes, clinical directors, managers, and implementers may consider using the current version of the toolkit for implementing iCBT services. The toolkit is freely accessible and may improve the outcome of local staff-driven implementation activities.

A temporal effect became apparent, pointing toward a small decline in normalization scores (total and of the underlying 4 constructs) in the IAU condition. The ItFits-toolkit partially canceled out this negative trend. This is surprising as, following the principles of NPT, we expected the normalization scores to increase when implementers and service providers engaged and worked to implement the iCBT services. Although speculative, this decline in normalization may be related to the complexity of iCBT services for service providers to deliver iCBT to their patients. Approximately three-quarters of the service deliverers were inexperienced in delivering iCBT before the study. As service providers started to spend more time delivering iCBT services, they might have gained a better understanding of its complexity and changes required to successfully integrate and embed the service in their routine practice, resulting in a decline in normalization scores. Similarly, gaining experience with providing iCBT services while at the same time being confronted with considerable patient attrition rates might have impacted the perception of service providers toward implementing the iCBT services. Furthermore, the possibly waning enthusiasm of service providers and implementers toward the research conducted and being part of a large-scale international research project might have influenced implementation outcomes. More research and debate are required to fully understand the theoretical implications of these findings.

For the secondary outcome, and although there are differences between organizations and the toolkit had no effect on service uptake, the high treatment dropout rate (72.6%) should be noted. The debate on dropout, adherence, and treatment completion was unsettled, and definitions differed greatly. A systematic review of dropout rates in research trials investigating the effectiveness of guided and unguided iCBT ranges from 0% to 78%, with an average of 21% stopping treatment early [66]. Other reviews of adherence found that, on average, approximately 61% to 65% of the patients complete their guided iCBT treatment [67,68]. Although we do not know why patients in our study stopped their treatment prematurely, it might have to do with the nature of their mental health problems such as chronicity or comorbidity [66], because they experienced the treatment as less beneficial to them [69], or because they recovered earlier than expected and did not require full exposure to the treatment [70]. In addition, adherence to an iCBT treatment is considered to be higher in a research setting than under routine care conditions [71]. In this study, the iCBT services were provided following routine care procedures and guidelines.

### Strengths and Limitations

This study was one of the first large-scale international collaborative research projects in which the primary focus was to use implementation science approaches to contribute to the implementation of iCBT services in routine mental health care. A strength of this trial was its high ecological validity. We managed to study a diverse group of implementers and iCBT service providers that were representative of routine care in 12 mental health service organizations in 8 European countries and Australia. Representing routine care mental health service delivery practice, the way in which mental health services were operationalized and delivered, including the clinical focus, guidance modalities, technical platform, and availability of mental health professionals and their experience with cognitive behavioral therapy, varied among the mental health service delivery organizations. A number of unforeseen events, ranging from internal staff turnover to changing legislation and reimbursement models, and natural disasters, such as bush fires, earthquakes, and the COVID-19 pandemic, occurred as they did. All available data were used, and by applying a pragmatic stepped-wedge cluster randomized controlled trial study design with repeated measures and a psychometrically validated implementation outcome measure, the study allowed for these variations to provide an accurate representation of real-world implementation practice. By randomizing the moment of introduction of the ItFits-toolkit in the implementation of mental health organizations, both conditions had an equal chance of being exposed to the events that occurred during the trial. Another strength was the systematic execution of the multisite study protocol within budget and time and with a centralized DCS providing high data quality [31]. Nevertheless, the findings need to be interpreted with care and are indicative of whether the conceptualization and operationalization of the tailored implementation as packaged in the ItFits-toolkit is a feasible idea.

Some methodological limitations should be considered when interpreting the results. One aspect was that IAU activities cannot be undone once embarked upon. The results might be influenced by carryover effects and intervention lag effects within the service delivery organizations. Therefore, it was likely that the findings originated from the ItFits-toolkit plus usual implementation activities. Likewise, the effects of the ItFits-toolkit might have become apparent beyond the data collection period. A second methodological limitation relates to outcome normalization, as measured by the NoMAD. This questionnaire was developed with precision and methodological rigor [45,72] and has been psychometrically validated in various studies in various settings [46-48]. However, these studies have used cross-sectional samples, and the psychometric sensitivity of NoMAD to longitudinal change is yet to be explored. In addition, and although speculative, regression to the mean in the primary outcome might have occurred as there was no option to rate items as “not applicable.” Respondents might have answered neutrally, whereas in reality, they experienced that some items were not relevant to their perception of the situation at that moment. Another factor that might have led to an underestimation of the effect was that some implementers had a background in research or were practicing research and that some had prior experience in implementing iCBT services. This experience might, for example, have affected adherence to some principles of the toolkit such as “being different” in implementing the iCBT service. Similarly, participating in a large-scale international research project designed to address implementation issues might have influenced implementers in their knowledge of and setting priorities in their implementation work. The forthcoming process evaluation will shed light on how implementers used the toolkit and can challenge these speculations.

### Future Research

The findings raised several new research questions. First, the effect was small. Depending on the research question and context, a small effect can be of importance to informing implementation processes. How this effect size should be interpreted in terms of practical improvement of implementation outcomes is yet to be determined. One direction of thought is that the toolkit supported organizational learning by systematically designing and applying evidence-informed implementation strategies over time to manage complex systems of change. Second, to optimize the effectiveness of the ItFits-toolkit, a dismantling study can be used to determine which components of self-guided tailored implementation contributed most to the outcomes of the ItFits-toolkit. A 3-phase Multiphase Optimization Strategy [73] approach using a factorial design might be a good way to quantitatively identify the most economical and effective combination of tailoring components that provide the best implementation outcomes. Third, implementation work is dynamic, takes time, and is context specific. The outcome measures used in this study showed little variation over time. Moreover, the normalization levels found in this study declined over time. This requires further discussion in theory development (NPT) and verification of the NoMAD with other instruments measuring implementation outcomes longitudinally and their sensitivity to change over time.

### Conclusions

The ItFits-toolkit had a small significant effect on normalization levels in mental health service providers. The toolkit did not change the uptake of iCBT by patients, and implementers did not spend more time using the toolkit in comparison with their usual ways of implementing iCBT services. Implementers generally regarded the ItFits-toolkit as usable and were satisfied with it. Although these findings are in line with expert-driven models of tailored implementation, they warrant modesty regarding the effectiveness of self-guided tailored implementation. Nevertheless, there is practical utility for implementers and clinical decision makers in self-guided tailored implementation of iCBT services in routine mental health care using the ItFits-toolkit.

## Appendix

Multimedia Appendix 1 Primary outcome information—Normalization Measure Development.

Multimedia Appendix 2 Secondary outcome—uptake and effort.

Multimedia Appendix 3 Secondary outcome—effort.

Multimedia Appendix 4 CONSORT-eHEALTH checklist (V 1.6.2).

## Abbreviations

CSQ: Client Satisfaction Questionnaire
DCS: Data Collection System
FTE: full-time equivalent
GP: general practitioner
IAU: implementation-as-usual
iCBT: internet-based cognitive behavioral therapy
IMA: ImpleMentAll
ItFits-toolkit: integrated theory-based Framework for intervention tailoring strategies toolkit
LMM: linear mixed-effects modeling
NoMAD: Normalization Measure Development
NPT: Normalization Process Theory
SUS: System Usability Scale
TIDieR: Template for Intervention Description and Replication
VAS: Visual Analog Scale
