# Supplementary material for: Effectiveness of Self-guided Tailored Implementation Strategies in Integrating and Embedding Internet-Based Cognitive Behavioral Therapy in Routine Mental Health Care: Results of a Multicenter Stepped-Wedge Cluster Randomized Trial
Source: J Med Internet Res. 2023 Feb 3;25:e41532. doi: 10.2196/41532 (PMC9938445; doi:10.2196/41532)
Supplement: Multimedia Appendix 1 [file jmir_v25i1e41532_app1.docx]

# Annex 1: Detailed information primary outcome: Normalisation

Scale and subscale scores NoMAD per wave and organisation

| **Organisation** | **Statistic** | **NoMAD score** | | | | | | | | | | | **Subscales (pooled)** | | | |
| --- | --- | --- | --- | --- | --- | --- | --- | --- | --- | --- | --- | --- | --- | --- | --- | --- |
|  |  | **Pooled** | **Wave 1** | **Wave 2** | **Wave 3** | **Wave 4** | **Wave 5** | **Wave 6** | **Wave 7** | **Wave 8** | **Wave 9** | **Wave 10** | **CO** | **CP** | **CA** | **RM** |
| IMA0101 | n | 236 | 21 | 25 | 27 | 22 | 23 | 23 | 27 | 24 | 23 | 21 | 236 | 236 | 236 | 236 |
|  | Mean | 3.72 | 3.86 | 3.67 | 3.65 | 3.80 | 3.74 | 3.66 | 3.74 | 3.62 | 3.75 | 3.74 | 3.74 | 3.92 | 3.57 | 3.75 |
|  | SD | 0.82 | 0.54 | 0.99 | 0.91 | 0.81 | 0.90 | 0.77 | 0.76 | 0.81 | 0.80 | 0.93 | 1.00 | 1.01 | 0.68 | 0.91 |
|  | Min | 1.20 | 2.20 | 1.20 | 1.20 | 1.20 | 1.20 | 1.20 | 1.20 | 1.20 | 1.20 | 1.20 | 1.00 | 1.00 | 1.57 | 1.00 |
|  | Max | 4.85 | 46.00 | 4.65 | 4.85 | 4.80 | 4.80 | 4.55 | 4.60 | 4.75 | 4.65 | 4.80 | 5.00 | 5.00 | 4.72 | 5.00 |
|  | Alpha | 0.97 |  | | | | | | | | | | 0.93 | 0.92 | 0.91 | 0.91 |
| IMA0201 | n | 146 | 9 | 19 | 9 | 18 | 19 | 18 | 17 | 9 | 13 | 15 | 146 | 146 | 146 | 146 |
|  | Mean | 3.74 | 3.79 | 3.49 | 3.90 | 3.73 | 3.62 | 3.84 | 3.94 | 3.78 | 3.70 | 3.77 | 3.86 | 4.04 | 3.51 | 3.74 |
|  | SD | 0.59 | 0.37 | 0.69 | 0.40 | 0.51 | 0.77 | 0.50 | 0.51 | 0.55 | 0.60 | 0.65 | 0.69 | 0.71 | 0.62 | 0.66 |
|  | Min | 1.25 | 3.25 | 2.10 | 3.35 | 2.80 | 1.25 | 2.90 | 3.00 | 3.00 | 2.85 | 2.40 | 1.00 | 1.00 | 1.57 | 1.20 |
|  | Max | 5.00 | 4.30 | 5.00 | 4.70 | 4.50 | 4.60 | 4.80 | 4.80 | 4.75 | 4.85 | 4.90 | 5.00 | 5.00 | 5.00 | 5.00 |
|  | Alpha | 0.93 |  | | | | | | | | | | 0.80 | 0.80 | 0.78 | 0.82 |
| IMA0301 | n | 134 | - | 7 | 16 | 17 | 17 | 16 | 18 | 16 | 14 | 13 | 134 | 134 | 134 | 134 |
|  | Mean | 3.64 | - | 3.44 | 3.78 | 3.77 | 3.60 | 3.59 | 3.54 | 3.49 | 3.70 | 3.73 | 3.66 | 2.83 | 3.51 | 3.64 |
|  | SD | 0.71 | - | 0.60 | 0.66 | 0.83 | 0.84 | 0.84 | 0.71 | 0.71 | 0.54 | 0.60 | 0.80 | 0.97 | 0.79 | 0.74 |
|  | Min | 1.35 | - | 2.60 | 2.55 | 1.35 | 1.95 | 1.65 | 2.15 | 2.10 | 2.75 | 2.85 | 1.00 | 1.00 | 1.50 | 1.20 |
|  | Max | 4.90 | - | 4.15 | 4.55 | 4.80 | 4.90 | 4.65 | 4.85 | 4.65 | 4.60 | 4.70 | 5.00 | 5.00 | 5.00 | 5.00 |
|  | Alpha | 0.91 |  | | | | | | | | | | 0.70 | 0.88 | 0.77 | 0.67 |
| IMA0302 | n | 429 | 36 | 46 | 44 | 45 | 46 | 43 | 42 | 43 | 42 | 42 | 429 | 429 | 429 | 429 |
|  | Mean | 3.76 | 3.94 | 3.84 | 3.76 | 3.79 | 3.81 | 3.79 | 3.65 | 3.63 | 3.71 | 3.68 | 3.83 | 4.10 | 3.62 | 3.62 |
|  | SD | 0.64 | 0.53 | 0.62 | 0.51 | 0.58 | 0.54 | 0.68 | 0.67 | 0.75 | 0.71 | 0.74 | 0.75 | 0.72 | 0.72 | 0.73 |
|  | Min | 1.20 | 2.95 | 2.20 | 2.30 | 2.70 | 2.85 | 1.20 | 1.60 | 1.20 | 2.10 | 1.20 | 1.00 | 1.00 | 1.57 | 1.00 |
|  | Max | 5.00 | 4.80 | 4.95 | 4.90 | 4.90 | 4.85 | 5.00 | 4.90 | 4.95 | 5.00 | 4.85 | 5.00 | 5.00 | 5.00 | 5.00 |
|  | Alpha | 0.93 |  | | | | | | | | | | 0.80 | 0.82 | 0.78 | 0.78 |
| IMA0401 | n | 171 | 7 | 22 | 24 | 21 | 17 | 19 | 14 | 16 | 15 | 16 | 171 | 171 | 171 | 171 |
|  | Mean | 3.95 | 3.99 | 3.91 | 3.83 | 3.93 | 3.77 | 3.93 | 3.84 | 3.92 | 4.18 | 4.28 | 3.90 | 4.24 | 3.80 | 3.94 |
|  | SD | 0.58 | 0.72 | 0.59 | 0.57 | 0.54 | 0.57 | 0.43 | 0.48 | 0.66 | 0.71 | 0.62 | 0.63 | 0.65 | 0.68 | 0.72 |
|  | Min | 2.40 | 2.70 | 2.90 | 2.85 | 2.95 | 2.40 | 2.75 | 2.70 | 2.80 | 2.80 | 3.00 | 2.00 | 2.25 | 1.86 | 1.80 |
|  | Max | 4.90 | 4.70 | 4.80 | 4.65 | 4.65 | 4.55 | 4.60 | 4.40 | 4.90 | 4.90 | 4.90 | 5.00 | 5.00 | 5.00 | 5.00 |
|  | Alpha | 0.91 |  | | | | | | | | | | 0.62 | 0.76 | 0.79 | 0.75 |
| IMA0501 | n | 374 | 24 | 39 | 40 | 40 | 42 | 40 | 40 | 36 | 38 | 35 | 374 | 374 | 374 | 374 |
|  | Mean | 3.11 | 3.28 | 3.33 | 3.05 | 3.09 | 2.90 | 2.87 | 3.13 | 3.20 | 3.16 | 3.19 | 3.24 | 3.37 | 3.00 | 2.94 |
|  | SD | 0.75 | 0.62 | 0.55 | 0.67 | 0.64 | 0.69 | 0.64 | 0.82 | 0.94 | 0.91 | 0.87 | 0.98 | 0.91 | 0.68 | 0.85 |
|  | Min | 1.20 | 2.00 | 1.70 | 1.20 | 1.75 | 1.30 | 1.20 | 1.20 | 1.20 | 1.20 | 1.20 | 1.00 | 1.00 | 1.29 | 1.00 |
|  | Max | 4.95 | 4.70 | 4.25 | 4.30 | 4.25 | 4.95 | 3.95 | 4.45 | 4.70 | 4.50 | 4.35 | 5.00 | 5.00 | 4.86 | 5.00 |
|  | Alpha | 0.94 |  | | | | | | | | | | 0.86 | 0.84 | 0.77 | 0.86 |
| IMA0502 | n | 195 | - | 22 | 23 | 24 | 20 | 24 | 17 | 23 | 22 | 20 | 195 | 195 | 195 | 195 |
|  | Mean | 3.39 | - | 3.53 | 3.48 | 3.47 | 3.19 | 3.35 | 3.51 | 3.18 | 3.50 | 3.27 | 3.46 | 3.65 | 3.34 | 3.20 |
|  | SD | 0.76 | - | 0.56 | 0.64 | 0.85 | 0.92 | 0.83 | 0.69 | 0.79 | 0.78 | 0.75 | 0.85 | 1.01 | 0.71 | 0.88 |
|  | Min | 1.30 | - | 2.65 | 2.10 | 1.45 | 1.35 | 1.30 | 1.80 | 1.60 | 1.40 | 1.60 | 1.00 | 1.00 | 1.57 | 5.00 |
|  | Max | 5.00 | - | 4.75 | 4.65 | 4.55 | 4.55 | 4.90 | 4.40 | 4.70 | 5.00 | 4.60 | 5.00 | 5.00 | 5.00 | 5.00 |
|  | Alpha | 0.93 |  | | | | | | | | | | 0.73 | 0.87 | 0.75 | 0.84 |
| IMA0601 | n | 209 | - | 22 | 25 | 24 | 24 | 22 | 24 | 23 | 23 | 22 | 209 | 209 | 209 | 209 |
|  | Mean | 3.80 | - | 3.66 | 3.68 | 3.75 | 3.73 | 3.92 | 3.84 | 3.90 | 3.95 | 3.82 | 3.85 | 3.97 | 3.69 | 3.79 |
|  | SD | 0.64 | - | 0.60 | 0.54 | 0.62 | 0.62 | 0.50 | 0.76 | 0.73 | 0.54 | 0.84 | 0.68 | 0.82 | 0.73 | 0.74 |
|  | Min | 1.30 | - | 1.45 | 1.80 | 1.75 | 1.95 | 2.95 | 1.75 | 1.40 | 3.00 | 1.30 | 1.25 | 1.00 | 1.14 | 1.00 |
|  | Max | 5.00 | - | 4.40 | 4.40 | 4.75 | 4.95 | 4.95 | 4.90 | 4.95 | 4.90 | 5.00 | 5.00 | 5.00 | 5.00 | 5.00 |
|  | Alpha | 0.93 |  | | | | | | | | | | 0.62 | 0.89 | 0.83 | 0.85 |
| IMA0701 | n | 200 | 14 | 21 | 21 | 21 | 21 | 21 | 21 | 20 | 20 | 20 | 200 | 200 | 200 | 200 |
|  | Mean | 4.05 | 3.94 | 4.16 | 4.04 | 3.98 | 4.09 | 4.00 | 4.03 | 4.18 | 4.00 | 4.06 | 4.10 | 4.39 | 4.00 | 3.81 |
|  | SD | 0.65 | 0.47 | 0.48 | 0.54 | 0.71 | 0.74 | 0.75 | 0.72 | 0.62 | 0.75 | 0.69 | 0.68 | 0.72 | 0.71 | 0.81 |
|  | Min | 1.60 | 3.25 | 2.75 | 2.95 | 2.70 | 2.60 | 1.60 | 2.15 | 3.00 | 2.10 | 3.00 | 2.00 | 1.00 | 1.57 | 1.00 |
|  | Max | 5.00 | 4.70 | 4.90 | 4.95 | 5.00 | 5.00 | 4.95 | 5.00 | 5.00 | 5.00 | 5.00 | 5.00 | 5.00 | 5.00 | 5.00 |
|  | Alpha | 0.93 |  | | | | | | | | | | 0.75 | 0.83 | 0.81 | 0.83 |
| IMA0801 | n | 367 | 35 | 42 | 42 | 42 | 33 | 34 | 40 | 36 | 33 | 30 | 367 | 367 | 367 | 367 |
|  | Mean | 3.72 | 3.64 | 3.71 | 3.58 | 3.75 | 3.70 | 3.69 | 3.75 | 3.75 | 3.78 | 3.86 | 3.87 | 4.08 | 3.47 | 3.65 |
|  | SD | 0.54 | 0.63 | 0.52 | 0.59 | 0.58 | 0.42 | 0.63 | 0.55 | 0.36 | 0.47 | 0.54 | 0.66 | 0.70 | 0.61 | 0.64 |
|  | Min | 1.20 | 1.35 | 2.25 | 1.20 | 1.20 | 2.90 | 1.20 | 2.50 | 3.15 | 2.95 | 2.90 | 1.00 | 1.00 | 1.57 | 1.00 |
|  | Max | 4.85 | 4.46 | 4.80 | 4.70 | 4.85 | 4.80 | 4.70 | 4.80 | 4.80 | 4.80 | 4.85 | 5.00 | 5.00 | 5.00 | 5.00 |
|  | Alpha | 0.89 |  | | | | | | | | | | 0.77 | 0.80 | 0.72 | 0.67 |
| IMA0802 | n | 238 | 1 | 35 | 26 | 29 | 28 | 27 | 25 | 22 | 22 | 23 | 238 | 238 | 238 | 238 |
|  | Mean | 3.11 | 3.30 | 3.36 | 3.25 | 3.11 | 3.13 | 3.14 | 3.07 | 2.86 | 2.90 | 2.95 | 3.28 | 3.34 | 2.99 | 2.95 |
|  | SD | 0.69 | - | 0.55 | 0.65 | 0.81 | 0.60 | 0.77 | 0.64 | 0.70 | 0.77 | 0.68 | 0.86 | 0.84 | 0.70 | 0.78 |
|  | Min | 1.20 | 3.30 | 1.50 | 1.20 | 1.35 | 1.90 | 1.60 | 1.80 | 1.30 | 1.60 | 1.50 | 1.00 | 1.00 | 1.29 | 1.00 |
|  | Max | 4.80 | 3.30 | 4.65 | 4.65 | 4.80 | 4.60 | 4.75 | 4.70 | 3.95 | 4.30 | 4.30 | 5.00 | 5.00 | 5.00 | 5.00 |
|  | Alpha | 0.94 |  | | | | | | | | | | 0.86 | 0.84 | 0.83 | 0.84 |
| IMA0901 | n | 185 | 19 | 17 | 18 | 19 | 19 | 18 | 18 | 19 | 19 | 19 | 185 | 185 | 185 | 185 |
|  | Mean | 4.32 | 4.42 | 4.38 | 4.30 | 4.28 | 4.22 | 4.36 | 4.39 | 4.29 | 4.34 | 4.28 | 4.29 | 4.63 | 4.10 | 4.42 |
|  | SD | 0.41 | 0.34 | 0.28 | 0.40 | 0.45 | 0.43 | 0.44 | 0.34 | 0.51 | 0.42 | 0.47 | 0.63 | 0.44 | 0.44 | 0.59 |
|  | Min | 3.05 | 3.60 | 4.00 | 3.40 | 3.25 | 3.25 | 3.30 | 3.60 | 3.05 | 3.45 | 3.15 | 2.50 | 2.75 | 2.57 | 2.60 |
|  | Max | 4.85 | 4.85 | 4.80 | 4.75 | 4.80 | 4.85 | 4.80 | 4.80 | 4.85 | 4.80 | 4.80 | 5.00 | 5.00 | 4.86 | 5.00 |
|  | Alpha | 0.89 |  | | | | | | | | | | 0.65 | 0.61 | 0.74 | 0.77 |


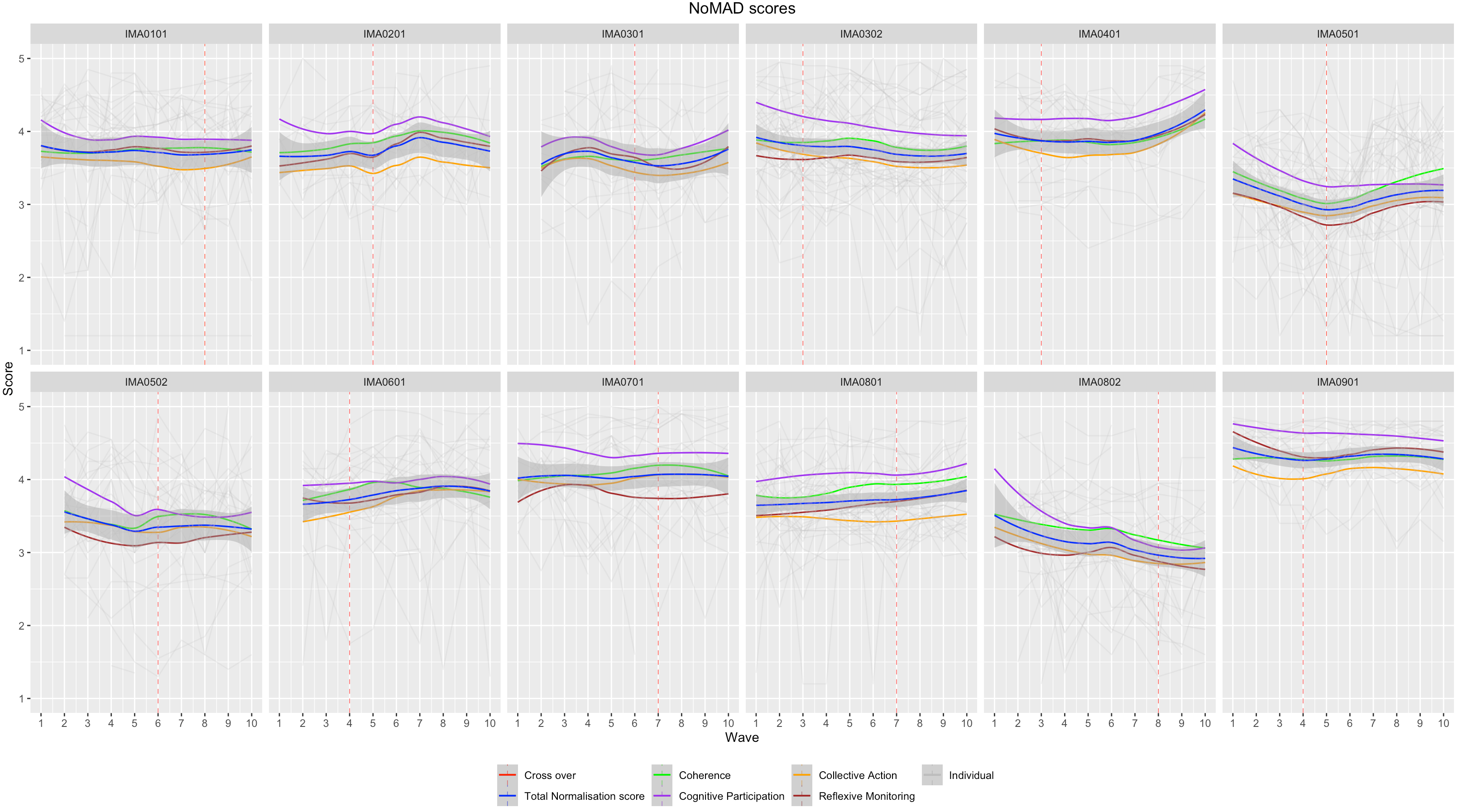


Figure: Mean NoMAD scale scorers, subscale scores, and underlying individual mental health service providers for each mental health service delivery organisation across time.

**Model specifications** (Hussey & Hughes (2007) in “pseudo R code”

Impact of intervention and wave on normalisation levels of all staff members

- Formula: NORM ~ factor(Wave) + Intervention + (1 | OrganisationID/StaffID)
- where: Intervention = .5 * (Wave > CrossOver) + .5 * (Wave > CrossOver + 1)
- Data: all data
- n_observations_ = 2884, n_groups (staff within org)_ = 445, n _org_ = 12
- REML criterion at convergence: 4319.6

*Scaled residuals*

| **Min** | **1Q** | **Median** | **3Q** | **Max** |
| --- | --- | --- | --- | --- |
| -6.07 | -0.44 | 0.02 | 0.49 | 3.81 |

*Random effects*

| **Groups** | **Name** | **Variance** | **SD** |
| --- | --- | --- | --- |
| StaffID:OrganisationID | (Intercept) | 0.25 | 0.50 |
| OrganisationID | (Intercept) | 0.11 | 0.33 |
| Residual | | 0.18 | 0.43 |

*Inter-cluster correlation (random variance explained by group membership)*

| ICC Staff | 0.58 |
| --- | --- |
| ICC Org | 0.37 |

*Fixed effects*

| **Measurement** | **Estimate** | **SD** | **95% CI** | **df** | ***t*** | ***p-value*** |
| --- | --- | --- | --- | --- | --- | --- |
| Wave 1 | 3.74 | 0.10 | 3.53, 3.95 | 14 | 35.94 | 0.00 |
| Wave 2 | 0.01 | 0.04 | -0.08, 0.09 | 2535 | 0.17 | 0.87 |
| Wave 3 | -0.07 | 0.04 | -0.15, 0.02 | 2532 | -1.59 | 0.11 |
| Wave 4 | -0.04 | 0.04 | -0.13, 0.04 | 2542 | -1.03 | 0.31 |
| Wave 5 | -0.13 | 0.04 | -0.22, -0.05 | 2548 | -3.03 | 0.00 |
| Wave 6 | -0.13 | 0.05 | -0.22, -0.04 | 2546 | -2.84 | 0.00 |
| Wave 7 | -0.11 | 0.05 | -0.20, -0.01 | 2553 | -2.17 | 0.03 |
| Wave 8 | -0.17 | 0.05 | -0.27, -0.07 | 2548 | -3.22 | 0.00 |
| Wave 9 | -0.13 | 0.06 | -0.24, -0.03 | 2547 | -2.41 | 0.02 |
| Wave 10 | -0.13 | 0.06 | -0.24, -0.02 | 2545 | -2.27 | 0.02 |
| ItFits = TRUE | 0.09 | 0.04 | 0.01, 0.16 | 2514 | 2.35 | 0.02 |

*Test temporal effects (H0 = all waves are equal 0)*

- Base model: NORM ~ Intervention + (1 | OrganisationID/StaffID)
- Interactional model: NORM ~ factor(Wave) + Intervention + (1 | OrganisationID/StaffID)

| **Model** | ***n*par** | **AIC** | **BIC** | **logLik** | **deviance** | **Chi^2^** | **Df** | ***p-value*** |
| --- | --- | --- | --- | --- | --- | --- | --- | --- |
| Base model | 5.00 | 4300.80 | 4330.70 | -2145.40 | 4290.80 |  |  |  |
| Interactional model | 14.00 | 4293.10 | 4376.70 | -2132.60 | 4265.10 | 25.695 | 9.00 | 0.002 |

*Effect Size using Cohen's* *d*

| **Method of standardisation** | ***d*** |
| --- | --- |
| Standardised on pooled SD | 0.12 |
| Standardised on SD wave 1 | 0.14 |
| Standardised on SD wave 10 | 0.11 |
