# Supplementary material for: Effectiveness of Self-guided Tailored Implementation Strategies in Integrating and Embedding Internet-Based Cognitive Behavioral Therapy in Routine Mental Health Care: Results of a Multicenter Stepped-Wedge Cluster Randomized Trial
Source: J Med Internet Res. 2023 Feb 3;25:e41532. doi: 10.2196/41532 (PMC9938445; doi:10.2196/41532)
Supplement: Multimedia Appendix 2 [file jmir_v25i1e41532_app2.docx]

# Annex 2: Detailed information Secondary outcome: Service Uptake

Cumulative uptake levels (and change in uptake levels) per wave and organisation. NA means Not Applicable.

| **Data set** | **Variable** | **Cumulative (𝚫)** | | | | | | | | | |
| --- | --- | --- | --- | --- | --- | --- | --- | --- | --- | --- | --- |
|  |  | **Wave 1** | **Wave 2** | **Wave 3** | **Wave 4** | **Wave 5** | **Wave 6** | **Wave 7** | **Wave 8** | **Wave 9** | **Wave 10** |
| Pooled | Referrals | 1608 (NA) | 1911 (303) | 2419 (508) | 3030 (611) | 3673 (643) | 4309 (636) | 4886 (577) | 5444 (558) | 6527 (558) | 7191 (664) |
|  | Completed | 280 (NA) | 335 (55) | 416 (81) | 503 (87) | 670 (167) | 790 (120) | 884 (94) | 1056 (172) | 1298 (242) | 1430 (132) |
|  | Not started | 34 | 50 | 73 | 144 | 159 | 208 | 175 | 176 | 275 | 131 |
|  | In treatment | 152 | 198 | 257 | 522 | 519 | 497 | 430 | 461 | 739 | 410 |
|  | Dropped out | 1,142 | 1,328 | 1,673 | 1,808 | 2,325 | 2,814 | 3,397 | 3,751 | 4,060 | 5,220 |
| IMA0101 | Referrals | 1 (NA) | 19 (18) | 35 (16) | 51 (16) | 79 (28) | 86 (7) | 121 (35) | 147 (26) | 167 (20) | 179 (12) |
|  | Completed | 0 (NA) | 0 (0) | 0 (0) | 3 (3) | 4 (1) | 6 (2) | 12 (6) | 14 (2) | 15 (1) | 15 (0) |
|  | Not started | 1 | 7 | 16 | 6 | 14 | 1 | 14 | 3 | - | 2 |
|  | In treatment | 0 | 12 | 19 | 6 | 14 | 6 | 18 | 8 | - | 4 |
|  | Dropped out | 0 | 0 | 0 | 36 | 47 | 73 | 77 | 122 | - | 158 |
| IMA0201 | Referrals | 0 (NA) | 33 (33) | 55 (22) | 77 (22) | 94 (17) | 112 (18) | 124 (12) | 138 (14) | 138 (0) | 149 (11) |
|  | Completed | 0 (NA) | 2 (2) | 2 (0) | 4 (2) | 4 (0) | 5 (1) | 6 (1) | 6 (6) | 6 (0) | 9 (3) |
|  | Not started | 0 | 2 | 0 | 1 | 0 | 1 | 0 | 0 | 0 | 0 |
|  | In treatment | 0 | 1 | 1 | 0 | 2 | 0 | 2 | 0 | 0 | 1 |
|  | Dropped out | 0 | 28 | 52 | 72 | 88 | 106 | 116 | 132 | 132 | 139 |
| IMA0301 | Referrals | 1285 (NA) | 1434 (149) | 1667 (233) | 2003 (336) | 2313 (310) | 2674 (361) | 2981 (307) | 3328 (347) | 4157 (829) | 4645 (488) |
|  | Completed | 206 (NA) | 232 (26) | 280 (48) | 336 (56) | 381 (45) | 409 (28) | 461 (52) | 508 (47) | 640 (132) | 728 (88) |
|  | Not started | 16 | 9 | 32 | 104 | 88 | 159 | 103 | 137 | 246 | 103 |
|  | In treatment | 98 | 109 | 44 | 188 | 186 | 174 | 149 | 184 | 491 | 186 |
|  | Dropped out | 965 | 1,084 | 1,311 | 1,375 | 1,658 | 1,932 | 2,268 | 2,499 | 2,780 | 3,628 |
| IMA0302 | Referrals | 10 (NA) | 14 (4) | 19 (5) | 24 (5) | 29 (5) | 30 (1) | 32 (2) | 32 (0) | 36 (4) | 40 (4) |
|  | Completed | 5 (NA) | 5 (0) | 10 (5) | 11 (1) | 14 (3) | 15 (1) | 16 (1) | 18 (2) | 22 (4) | 25 (3) |
|  | Not started | 0 | 1 | 0 | 0 | 0 | 1 | 0 | 0 | 0 | 0 |
|  | In treatment | 1 | 4 | 3 | 4 | 5 | 2 | 2 | 0 | 0 | 1 |
|  | Dropped out | 4 | 4 | 6 | 9 | 10 | 12 | 14 | 14 | 14 | 14 |
| IMA0401 | Referrals | 0 (NA) | 0 (0) | 132 (132) | 264 (132) | 413 (149) | 576 (163) | 696 (120) | 797 (101) | 881 (84) | 958 (77) |
|  | Completed | 0 (NA) | 0 (0) | 0 (0) | 0 (0) | 77 (77) | 136 (59) | 137 (1) | 223 (86) | 276 (53) | 301 (25) |
|  | Not started | 0 | 0 | 6 | 3 | 11 | 8 | 22 | 4 | 4 | 2 |
|  | In treatment | 0 | 0 | 126 | 251 | 210 | 242 | 179 | 203 | 130 | 116 |
|  | Dropped out | 0 | 0 | 0 | 10 | 115 | 190 | 358 | 367 | 471 | 539 |
| IMA0501 | Referrals | 20 (NA) | 32 (12) | 34 (2) | 39 95) | 47 (8) | 50 (3) | 63 (13) | 66 (3) | 66 (0) | 71 (5) |
|  | Completed | 4 (NA)) | 15 (11) | 20 (5) | 23 (3) | 27 (4) | 32 (5) | 41 (9) | 51 (10) | 54 (3) | 54 (0) |
|  | Not started | 4 | 4 | 1 | 0 | 0 | 0 | 2 | 3 | 0 | 0 |
|  | In treatment | 9 | 7 | 3 | 5 | 9 | 6 | 8 | 0 | 0 | 5 |
|  | Dropped out | 3 | 6 | 10 | 11 | 11 | 12 | 12 | 12 | 12 | 12 |
| IMA0502 | Referrals | 0 (NA) | 0 (0) | 0 (0) | 2 (2) | 2 (0) | 3 (1) | 14 (11) | 14 (0) | 14 (0) | 14 (0) |
|  | Completed | 0 (NA) | 0 (0) | 0 (0) | 0 (0) | 1 (1) | 1 (0) | 3 (2) | 4 (1) | 10 (6) | 10 (0) |
|  | Not started | 0 | 0 | 0 | 0 | 0 | 1 | 5 | 5 | 0 | 0 |
|  | In treatment | 0 | 0 | 0 | 1 | 0 | 0 | 5 | 4 | 0 | 0 |
|  | Dropped out | 0 | 0 | 0 | 1 | 1 | 1 | 1 | 1 | 4 | 4 |
| IMA0601 | Referrals | 0 (NA) | 9 (9) | 29 (20) | 50 (21) | 69 (19) | 78 (9) | 87 (9) | 89 (2) | 98 (9) | 100 (2) |
|  | Completed | 0 (NA) | 0 (0) | 1 (1) | 7 (6) | 14 (7) | 20 (6) | 23 (3) | 25 (2) | 31 (6) | 32 (1) |
|  | Not started | 0 | 2 | 5 | 12 | 15 | 10 | 10 | 10 | 8 | 8 |
|  | In treatment | 0 | 7 | 19 | 20 | 19 | 15 | 13 | 12 | 17 | 18 |
|  | Dropped out | 0 | 0 | 4 | 11 | 21 | 33 | 41 | 42 | 42 | 42 |
| IMA0701 | Referrals | 12 (NA) | 26 (14) | 41 (15) | 48 (7) | 79 (31) | 96 (17) | 115 (19) | 130 (15) | 136 (6) | 145 (9) |
|  | Completed | 1 (NA) | 5 (4) | 13 (8) | 15 (2) | 28 (13) | 36 (8) | 41 (5) | 49 (8) | 55 (6) | 57 (2) |
|  | Not started | 0 | 4 | 2 | 2 | 5 | 4 | 3 | 2 | 0 | 0 |
|  | In treatment | 5 | 8 | 2 | 5 | 15 | 7 | 13 | 7 | 4 | 6 |
|  | Dropped out | 6 | 9 | 24 | 26 | 31 | 49 | 58 | 72 | 77 | 82 |
| IMA0801 | Referrals | 280 (NA) | 315 (35) | 360 (45) | 408 (48) | 467 (59) | 510 (43) | 549 (39) | 584 (35) | 695 (111) | 748 (53) |
|  | Completed | 64 (NA) | 72 (8) | 83 (11) | 93 (10) | 108 (15) | 118 (10) | 129 (11) | 138 (9) | 169 (31) | 179 (10) |
|  | Not started | 13 | 15 | 11 | 17 | 21 | 16 | 13 | 10 | 11 | 14 |
|  | In treatment | 39 | 34 | 37 | 40 | 49 | 37 | 32 | 35 | 84 | 71 |
|  | Dropped out | 164 | 194 | 229 | 258 | 289 | 339 | 375 | 401 | 431 | 484 |
| IMA0802 | Referrals | 0 (NA) | 0 (0) | 0 (0) | 3 (3) | 5 (2) | 8 (3) | 8 (0) | 9 (1) | 11 (2) | 11 (0) |
|  | Completed | 0 (NA) | 0 (0) | 0 (0) | 0 (0) | 0 (0) | 0 (0) | 0 (0) | 0 (0) | 0 (0) | 0 (0) |
|  | Not started | 0 | 0 | 0 | 0 | 1 | 4 | 0 | 0 | 1 | 1 |
|  | In treatment | 0 | 0 | 0 | 3 | 1 | 0 | 2 | 2 | 1 | 0 |
|  | Dropped out | 0 | 0 | 0 | 0 | 3 | 4 | 6 | 7 | 9 | 10 |
| IMA0901 | Referrals | 0 (NA) | 29 (29) | 47 (18) | 61 (14) | 76 (15) | 86 (10) | 96 (10) | 110 (14) | 128 (18) | 131 (3) |
|  | Completed | 0 (NA) | 4 (4) | 7 (3) | 11 (4) | 12 (1) | 12 (0) | 15 (3) | 20 (5) | 20 (0) | 20 (0) |
|  | Not started | 0 | 6 | 0 | - | 4 | 3 | 3 | 2 | 6 | 1 |
|  | In treatment | 0 | 16 | 3 | - | 9 | 8 | 7 | 6 | 13 | 2 |
|  | Dropped out | 0 | 3 | 37 | - | 51 | 63 | 71 | 82 | 89 | 108 |


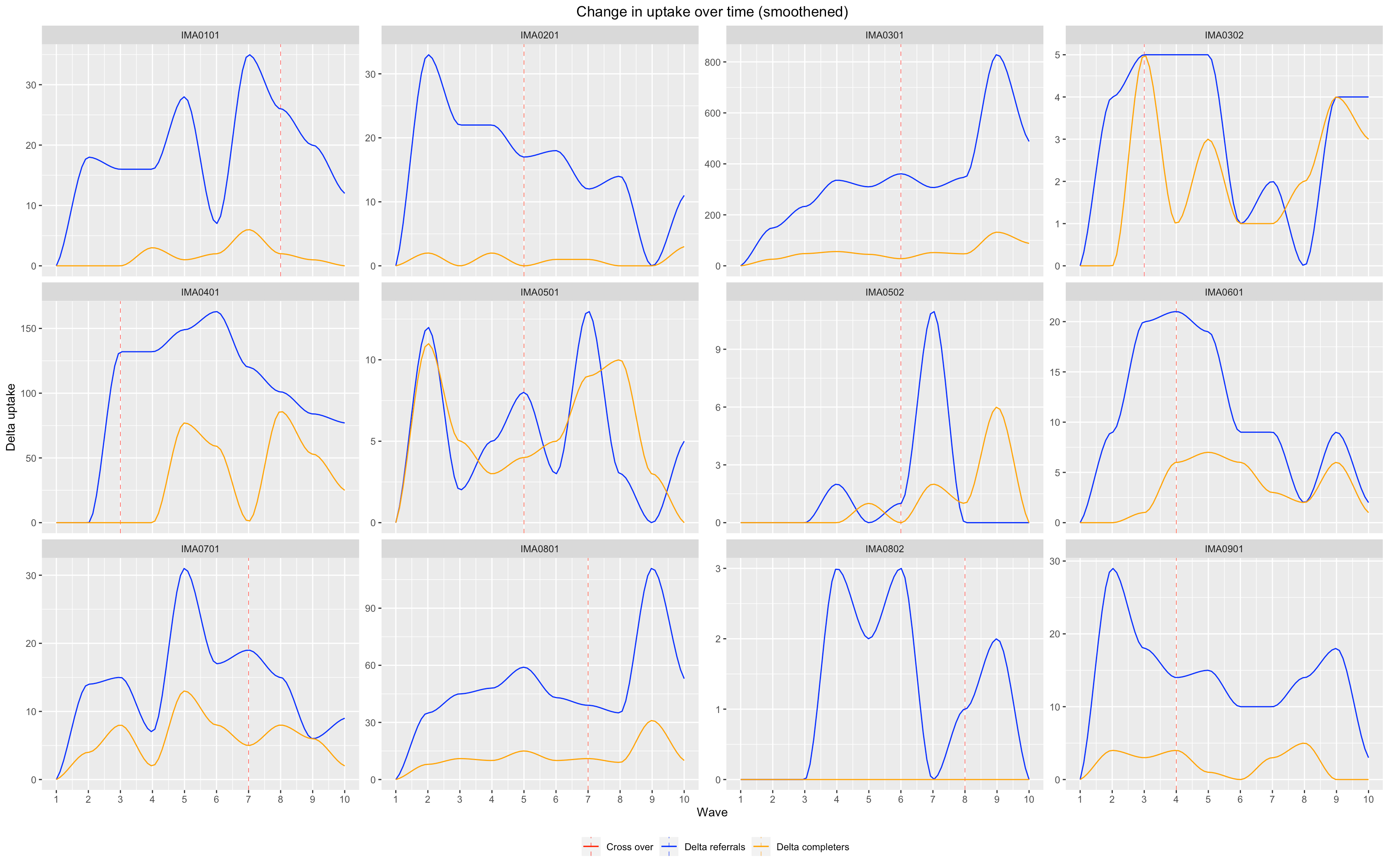
Figure: Change in uptake levels over time and for each mental health service delivery organisation.

**Model specifications** (Hussey & Hughes (2007) in “pseudo R code”.

Impact of intervention and wave on uptake (referrals) on organisational level

- Formula: deltaReferrals ~ factor(Wave) + Intervention + (1 + Wave | OrganisationID)
- where: Intervention = .5 * (Wave > CrossOver) + .5 * (Wave > CrossOver + 1)
- Data: all data (referrals)
- n_ovservations_ = 120, n _org_ = 12
- REML criterion at convergence: 1278.1

*Scaled residuals*

| **Min** | **1Q** | **Median** | **3Q** | **Max** |
| --- | --- | --- | --- | --- |
| -4.30 | -0.21 | -0.05 | 0.21 | 7.13 |

*Random effects*

| **Groups** | **Name** | **Variance** | **SD** |
| --- | --- | --- | --- |
| OrganisationID | (Intercept) | 8656 | 93.04 |
| Residual | | 4157 | 64.48 |

*Inter-cluster correlation (random variance explained by group membership)*

| ICC Org | 0.68 |
| --- | --- |

*Fixed effects*

| **Measurement** | **Estimate** | **SE** | **95% CI** | **df** | ***t*** | ***p-value*** |
| --- | --- | --- | --- | --- | --- | --- |
| Wave 1 | 0.00 | 32.68 | -64, 64 | 21.53 | 0.00 | 1.00 |
| Wave 2 | 25.25 | 26.32 | -24.34, 74.84 | 98.05 | 0.96 | 0.34 |
| Wave 3 | 42.33 | 26.32 | -7.26, 91.92 | 98.05 | 1.61 | 0.11 |
| Wave 4 | 49.12 | 26.42 | -0.64, 98.89 | 98.09 | 1.86 | 0.07 |
| Wave 5 | 48.21 | 27.16 | -2.95, 99.37 | 98.38 | 1.78 | 0.08 |
| Wave 6 | 44.04 | 28.58 | -9.79, 97.89 | 98.86 | 1.54 | 0.13 |
| Wave 7 | 35.54 | 30.59 | -22.07, 93.18 | 99.42 | 1.16 | 0.25 |
| Wave 8 | 30.37 | 33.08 | -31.93, 92.7 | 99.97 | 0.92 | 0.36 |
| Wave 9 | 70.54 | 35.95 | 2.84, 138.28 | 100.40 | 1.96 | 0.05 |
| Wave 10 | 33.83 | 37.50 | -36.79, 104.49 | 100.70 | 0.90 | 0.37 |
| ItFits = TRUE | 21.50 | 26.71 | -28.82, 71.8 | 102.90 | 0.81 | 0.42 |

*Test temporal effects (H0 = all waves are equal 0)*

- Base model: deltaReferrals ~ Intervention + (1 | OrganisationID)
- Interactional model: deltaReferrals ~ factor(Wave) + Intervention + (1 | OrganisationID)

| **Model** | ***n*par** | **AIC** | **BIC** | **logLik** | **deviance** | **Chi^2^** | **Df** | ***p-value*** |
| --- | --- | --- | --- | --- | --- | --- | --- | --- |
| Base model | 4.00 | 1382.20 | 1393.30 | -687.09 | 1374.20 |  |  |  |
| Interactional model | 13.00 | 1391.90 | 1428.20 | -682.96 | 1365.90 | 8.27 | 9 | 0.51 |
