# Supplementary material for: Effectiveness of Self-guided Tailored Implementation Strategies in Integrating and Embedding Internet-Based Cognitive Behavioral Therapy in Routine Mental Health Care: Results of a Multicenter Stepped-Wedge Cluster Randomized Trial
Source: J Med Internet Res. 2023 Feb 3;25:e41532. doi: 10.2196/41532 (PMC9938445; doi:10.2196/41532)
Supplement: Multimedia Appendix 3 [file jmir_v25i1e41532_app3.docx]

# Annex 3: Detailed information Secondary outcome: Effort

Formula applied to calculate Full-time Equivalent pooled effort: 20,277.5 / 3 = 6,759.2 hours per year. Assuming one FTE equals 1,650 hours gives that 6,759.2 / 1,650 = 4.09 FTE per year is spent on implementation work. Note: the total given concerns the sum of effort of the implementation core teams in all mental health service delivery organisations over the whole study period.

| **Data set** | **Hours** | | | | | | | | | | | |
| --- | --- | --- | --- | --- | --- | --- | --- | --- | --- | --- | --- | --- |
|  | **Wave 1** | **Wave 2** | **Wave 3** | **Wave 4** | **Wave 5** | **Wave 6** | **Wave 7** | **Wave 8** | **Wave 9** | **Wave 10** | **Cummulative** | **FTE (per year)^1^** |
| Total | 1,371 | 1,257 | 2,191 | 2,371 | 2,795 | 2,266 | 2,355 | 2,012 | 1,836 | 1,824 | 20,278 | 4.92 |
| IMA0101 | 0 | 39 | 112 | 400 | 250 | 250 | 250 | 250 | 150 | 250 | 1,951 | 0.47 |
| IMA0201 | 53 | 61 | 29 | 26 | 20 | 25 | 43 | 27 | 11 | 23 | 318 | 0.08 |
| IMA0301 | 112 | 62 | 256 | 279 | 192 | 171 | 192 | 124 | 165 | 68 | 1,621 | 0.39 |
| IMA0302 | 226 | 238 | 273 | 454 | 454 | 454 | 454 | 446 | 459 | 459 | 3,917 | 0.95 |
| IMA0401 | 0 | 0 | 0 | 18 | 52 | 0 | 8 | 10 | 0 | 0 | 88 | 0.02 |
| IMA0501 | 10 | 30 | 24 | 20 | 24 | 24 | 24 | 24 | 24 | 24 | 228 | 0.06 |
| IMA0502 | 0 | 36 | 24 | 20 | 0 | 20 | 30 | 30 | 30 | 30 | 220 | 0.05 |
| IMA0601 | 0 | 0 | 5 | 56 | 210 | 225 | 225 | 250 | 225 | 225 | 1,421 | 0.34 |
| IMA0701 | 449 | 234 | 248 | 220 | 187 | 211 | 236 | 256 | 160 | 208 | 2,409 | 0.58 |
| IMA0801 | 104 | 416 | 442 | 442 | 442 | 442 | 442 | 442 | 442 | 442 | 4,056 | 0.98 |
| IMA0802 | 36 | 71 | 97 | 58 | 118 | 63 | 70 | 27 | 44 | 29 | 613 | 0.15 |
| IMA0901 | 381 | 70 | 681 | 378 | 846 | 381 | 381 | 126 | 126 | 66 | 3,436 | 0.83 |

1. Assumed number of hours for a yearly FTE productive position is 1,650.


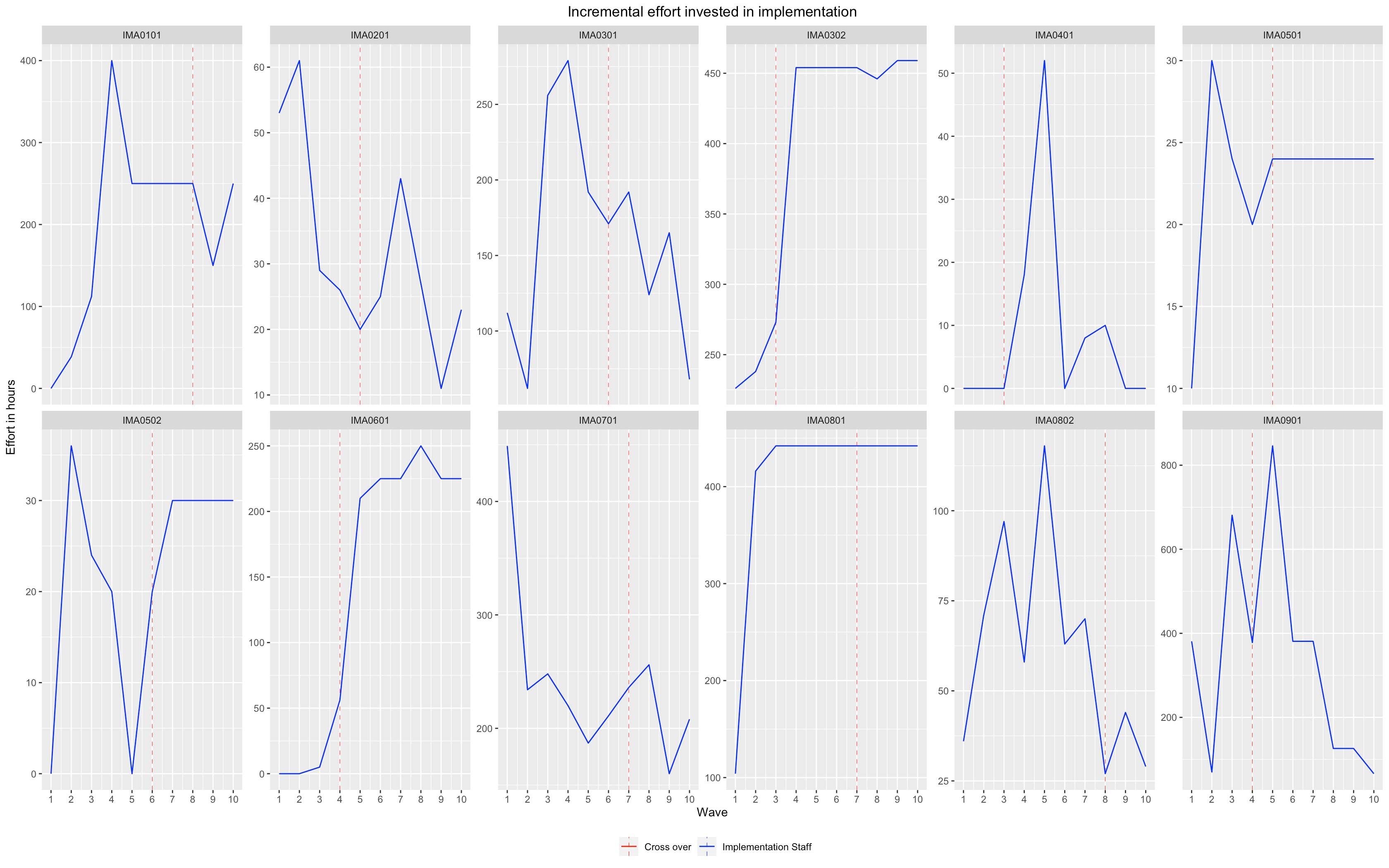
Figure: Effort spent by implementation teams during IAU and when using the ItFits-toolkit across time.

**Model specifications** (Hussey & Hughes (2007) in “pseudo R code”.

Impact of intervention and wave on uptake (referrals) on organisational level

- Formula: iEffort ~ factor(Wave) + Intervention + (1 + Wave | OrganisationID)
- where: Intervention = .5 * (Wave > CrossOver) + .5 * (Wave > CrossOver + 1)
- Data: all data (referrals)
- n_ovservations_ = 120, n _org_ = 12
- REML criterion at convergence: 1374.8

*Scaled residuals*

| **Min** | **1Q** | **Median** | **3Q** | **Max** |
| --- | --- | --- | --- | --- |
| -2.45 | -0.36 | -0.06 | 0.41 | 4.40 |

*Random effects*

| **Groups** | **Name** | **Variance** | **SD** |
| --- | --- | --- | --- |
| OrganisationID | (Intercept) | 21045 | 145.10 |
| Residual | | 4157 | 10091 |

*Inter-cluster correlation (random variance explained by group membership)*

| ICC Org | 0.68 |
| --- | --- |

*Fixed effects*

| **Measurement** | **Estimate** | **SE** | **95% CI** | **df** | ***t*** | ***p-value*** |
| --- | --- | --- | --- | --- | --- | --- |
| Wave 1 | 114.25 | 50.94 | 14.48, 214.02 | 21.51 | 2.24 | 0.04 |
| Wave 2 | -9.54 | 41.01 | -86.8, 67.72 | 98.05 | -0.23 | 0.82 |
| Wave 3 | 68.33 | 41.01 | -8.93, 145.6 | 98.05 | 1.67 | 0.10 |
| Wave 4 | 79.51 | 41.16 | 1.97, 157.05 | 98.09 | 1.93 | 0.06 |
| Wave 5 | 107.20 | 42.31 | 27.49, 186.91 | 98.38 | 2.53 | 0.01 |
| Wave 6 | 55.47 | 44.53 | -28.4, 139.36 | 98.86 | 1.25 | 0.22 |
| Wave 7 | 55.24 | 47.66 | -34.53, 145.03 | 99.42 | 1.16 | 0.25 |
| Wave 8 | 19.01 | 51.54 | -78.06, 116.11 | 99.96 | 0.37 | 0.71 |
| Wave 9 | -3.30 | 56.01 | -108.79, 102.22 | 100.45 | -0.06 | 0.95 |
| Wave 10 | -8.13 | 58.43 | -118.16, 101.95 | 100.66 | -0.14 | 0.89 |
| ItFits = TRUE | 45.88 | 41.62 | -32.52, 124.24 | 102.93 | 1.10 | 0.27 |

*Test temporal effects (H0 = all waves are equal 0)*

- Base model: iEffort ~ Intervention + (1 | OrganisationID)
- Interactional model: iEffort ~ factor(Wave) + Intervention + (1 | OrganisationID)

| **Model** | ***n*par** | **AIC** | **BIC** | **logLik** | **deviance** | **Chi^2^** | **Df** | ***p-value*** |
| --- | --- | --- | --- | --- | --- | --- | --- | --- |
| Base model | 4.00 | 1497.70 | 1508.80 | -744.85 | 1489.70 |  |  |  |
| Interactional model | 13.00 | 1498.30 | 1534.60 | -736.17 | 1472.30 | 17.35 | 9.00 | 0.04 |
