# Supplementary material for: Effectiveness of Self-guided Tailored Implementation Strategies in Integrating and Embedding Internet-Based Cognitive Behavioral Therapy in Routine Mental Health Care: Results of a Multicenter Stepped-Wedge Cluster Randomized Trial
Source: J Med Internet Res. 2023 Feb 3;25:e41532. doi: 10.2196/41532 (PMC9938445; doi:10.2196/41532)
Supplement: Multimedia Appendix 4 [file jmir_v25i1e41532_app4.pdf]

CONSORT-EHEALTH Checklist V1.6.2 Report	Manuscript Number	41532
**(based on CONSORT-EHEALTH V1.6), available at [http://tinyurl.com/consort-ehealth-v1-6].**		
**Date completed**		
1/16/2023 7:17:38		
**by**		
Christiaan Vis		
Effectiveness of Self-guided Tailored Implementation Strategies in Integrating and Embedding Internet-Based Cognitive Behavioral Therapy in Routine Mental Health Care: Results of a Multicenter Stepped-Wedge Cluster Randomized Trial		
TITLE		
**1a-i) Identify the mode of delivery in the title**		
Described in introduction: "As part of the Horizon 2020 ImpleMentAll (IMA) project [31], the ItFits-toolkit was specifically designed as a web-based self-guided tool for local implementers". Detailed in methods section.		
**1a-ii) Non-web-based components or important co-interventions in title**		
The toolkit provides suggestions to work with stakeholders; eg. brainstorming etc. This is explained in the methods section describing the intervention (ItFits-toolkit).		
**1a-iii) Primary condition or target group in the title**		
Title includes: "Self-guided Tailored Implementation Strategies ..."		
ABSTRACT		
**1b-i) Key features/functionalities/components of the intervention and comparator in the METHODS section of the ABSTRACT**		
"The self-guided integrated theory-based Framework for intervention tailoring strategies toolkit (ItFits-toolkit) supports local implementers in developing tailored implementation strategies. Tailoring involves identifying local barriers; matching selected barriers to implementation strategies; developing an actionable work plan; and applying, monitoring, and adapting where necessary."		
**1b-ii) Level of human involvement in the METHODS section of the ABSTRACT**		
"The self-guided integrated theory-based Framework for intervention tailoring strategies toolkit (ItFits-toolkit) supports local implementers in developing tailored implementation strategies."		
**1b-iii) Open vs. closed, web-based (self-assessment) vs. face-to-face assessments in the METHODS section of the ABSTRACT**		
"The primary outcome was the normalization of iCBT delivery by service providers (therapists, referrers, IT developers, and administrators), which was measured with the Normalization Measure Development as a proxy for implementation success."		
**1b-iv) RESULTS section in abstract must contain use data**		
"In total, 456 mental health service providers were included in this study."		
**1b-v) CONCLUSIONS/DISCUSSION in abstract for negative trials**		
Not applicable; not a negative trial		
INTRODUCTION		
**2a-i) Problem and the type of system/solution**		
Problem of implementation; lacking expectations, barriers, and issue of complexity and context are explained in detail in the introduction.		
**2a-ii) Scientific background, rationale: What is known about the (type of) system**		
Concept of tailored implementation is explained in detail in the introduction.		
**Does your paper address CONSORT subitem 2b?**		
"In this study, we examined the effectiveness of the ItFits-toolkit and answered the following research question: does the use of the ItFits-toolkit lead to better outcomes than implementation-as-usual (IAU) in implementing iCBT services in routine mental health care?" And: "We hypothesized that ItFits-toolkit use would be associated with increased normalization of iCBT services into practice. Parallel to this effectiveness study, an in-depth qualitative process evaluation was conducted, focusing on engagement, embedding, and integration of the ItFits-toolkit by implementers [33,34]."		
METHODS		
**3a) CONSORT: Description of trial design (such as parallel, factorial) including allocation ratio**		
Main design: A multicenter trial was conducted in Albania, Australia, Denmark, France, Germany, Italy, Kosovo, the Netherlands, and Spain. This study was conducted between March 2018 and March 2021. The study protocol is published elsewhere [31]." and: "A stepped-wedge cluster randomized controlled trial design was applied [35]. The main design principles are shown in Figure 1. Over a period of 30 months, the ItFits-toolkit was sequentially rolled out into 6 groups of 12 organizations (clusters). The clusters were randomly allocated with an interval of 3 months, at which the clusters crossed over from the control condition (IAU) to the experimental condition (ItFits-toolkit). Data were collected in 10 waves with a 3-month interval period (waves 1-10) to strike a balance between measuring change over time and the measurement burden imposed on the study participants."		
**3b) CONSORT: Important changes to methods after trial commencement (such as eligibility criteria), with reasons**		
No changes were made after the trial started.		
**3b-i) Bug fixes, Downtimes, Content Changes**		
No changes were made; system was tested rigorously prior to starting the study. System worked as designed and expected.		
**4a) CONSORT: Eligibility criteria for participants**		
Detailed in methods section: "Two types of participants were included in the study: (1) implementers, that is, local staff who facilitated the implementation of the iCBT service, and (2) mental health service providers such as therapists who were involved in iCBT service delivery.<br><br>For each of the 12 organizations, a team of up to 5 staff members was appointed as implementers. One team member was appointed as the implementation lead and coordinated the work. Implementers were directly involved in the development, coordination, and execution of local implementation activities such as designing and distributing iCBT information leaflets or developing training materials for referrers and therapists. Implementers were not required to have prior experience in, or specialist knowledge of, implementing iCBT services, but were expected to have working knowledge of the service they were implementing. Implementers could have different functions or roles in the organization, that is, manager, researcher, or clinician. Implementers were expected to have a proficient command of the English language to be able to use the ItFits-toolkit.<br><br>Mental health service providers were eligible to be included if they had a distinct role in delivering iCBT to patients, including clinicians, psychologists, GPs, psychiatrists, psychiatric nurses, staff in a supporting role (eg, administrators), or IT professionals involved in the operation of the technical aspects of the iCBT services."		
**4a-i) Computer / Internet literacy**		
Not relevant/ no criterium		
**4a-ii) Open vs. closed, web-based vs. face-to-face assessments:**		
Participants (implementers; service deliverers/staff) were recruited by direct mailing: "Local research teams recruited both implementers and service providers with the support of a central research team overseeing the trial. To avoid contamination between the implementation team and the target population of the toolkit, participants could not act as both implementers and service providers."		
**4a-iii) Information giving during recruitment**		
Participants received information about the study that was included in the direct mailing.		
**4b) CONSORT: Settings and locations where the data were collected**		
Detailed in methods section, including: "A total of 12 mental health service delivery organizations in 9 countries were included in the study (Table 1)."		
**4b-i) Report if outcomes were (self-)assessed through online questionnaires**		
We made use of a centralised data collection system: "Nested within the service delivery organizations, data were collected from implementers and service providers. Some data and outcomes (demographics, satisfaction, and usability) were collected once, whereas data on the primary and secondary outcomes were assessed every 3 months (Table 2). All questionnaires were translated using a forward-backward translation procedure [31]. All data were collected through a secure web-based central Data Collection System (DCS), which allowed a standardized and structured data collection process. The DCS was developed specifically for this study and designed to prevent missing values or false entries and to enable automatic anonymization."		
**4b-ii) Report how institutional affiliations are displayed**		
NA. this was a study amongst implementers and service deliverers.		
**5) CONSORT: Describe the interventions for each group with sufficient details to allow replication, including how and when they were actually administered**		
**5-i) Mention names, credential, affiliations of the developers, sponsors, and owners**		
Toolkit was built from scratch by the consortium. NA		
**5-ii) Describe the history/development process**		

"ItFits-toolkit was built from scratch before the start of recruitment to the trial and underwent various rounds of conceptual and technical piloting with various user groups<br>representing the perspectives of implementers, clinical stakeholders, and researchers. More information regarding the toolkit is available on the project website [43] and in the study<br>protocol [31]. This toolkit is freely accessible [44]."		
**5-iii) Revisions and updating**		
No changes made during study. Afterwards and prior to publishing the toolkit was adapted following learnings from the study, including more flexible navigation within the toolkit and support to run the toolkit in multiple-languages/instances.		
**5-iv) Quality assurance methods**		
Quality was assured through multiple levels of involvement of researchers: local teams were responsible for data collection, a central team provided guidance and conducted the analysis with consultation of an internal scientific advisory board and an external advisory board.		
**5-v) Ensure replicability by publishing the source code, and/or providing screenshots/screen-capture video, and/or providing flowcharts of the algorithms used**		
Design, contents and architecture of the toolkit are delivered to the European Commission by the ImpleMentAll consortium as formal deliverables. These ara available through the Commission's repository and the project website: www.implementall.eu.		
**5-vi) Digital preservation**		
www.itfits-toolkit.com; www.implementall.eu and repository of H2020 projects of the European commission		
**5-vii) Access**		
Access to the implementation toolkit was free.		
**5-viii) Mode of delivery, features/functionalities/components of the intervention and comparator, and the theoretical framework**		
Conceptual background is tailored implementation and the normalisation process theory. This is explained in the trial protocol paper and the methods section of current manuscript in detail.		
**5-ix) Describe use parameters**		
Implementers (ie. ItFits-toolkit users) were free to use the toolkit as they required (following concept of tailoring). However, for trial purposes, we asked them to go through a full cycle of the toolkit at least once in a time period of maximal 6 months: "A period of 6 months was chosen as the exposure period to balance the practical and financial feasibility of the study, with realistic opportunities for implementers to gain experience with the toolkit and being exposed to the core components of the toolkit. Adequate exposure to the ItFits-toolkit was defined as the implementers completing modules 1, 2, and 3 within the exposure period (Figure 2)."		
**5-x) Clarify the level of human involvement**		
The central trial team provided technical assistance to the implementers during exposure period: "At crossover (Figure 1), the implementers received access to the toolkit following an introductory training. The training covered the ItFits-toolkit working principles and technical instructions to get started." and: "During the exposure period, the sites received technical support in the form of monthly conference calls. As with the introductory training, the calls were limited to the technical use aspects of the toolkit and did not address any specific implementation advice such as which barriers to address or which strategy to use. The introductory training and calls were provided by members of the central research team involved in the development of the ItFits-toolkit."		
**5-xi) Report any prompts/reminders used**		
NA. Implementers were not reminded actively as we did not want to infringe their flexibility to use the toolkit as they required (following concept of tailoring).		
**5-xii) Describe any co-interventions (incl. training/support)**		
The central trial team provided technical assistance to the implementers during exposure period: "At crossover (Figure 1), the implementers received access to the toolkit following an introductory training. The training covered the ItFits-toolkit working principles and technical instructions to get started." and: "During the exposure period, the sites received technical support in the form of monthly conference calls. As with the introductory training, the calls were limited to the technical use aspects of the toolkit and did not address any specific implementation advice such as which barriers to address or which strategy to use. The introductory training and calls were provided by members of the central research team involved in the development of the ItFits-toolkit."		
**6a) CONSORT: Completely defined pre-specified primary and secondary outcome measures, including how and when they were assessed**		
Outcome measures were defined and did not change during the trail. They are described in detail in the protocol paper and in the current manuscript: "The primary outcome was the degree of normalization among service providers." [...] and " To complement the primary outcome, we assessed the effectiveness of the ItFits-toolkit using measures of uptake of<br>the iCBT service by patients (referral and completed treatments with adequate exposure levels) and implementation efficiency (operationalized by hours spent by implementers). These were used as secondary outcomes."		
**6a-i) Online questionnaires: describe if they were validated for online use and apply CHERRIES items to describe how the questionnaires were designed/deployed**		
Questionaire for the primary outcome was validated: "Normalization was measured using the Normalization Measure Development (NoMAD) questionnaire [45,46]. The NoMAD is a brief self-reported questionnaire with 20 items addressing the 4 generative mechanisms involved in implementation processes, as conceptualized by NPT: coherence, cognitive participation, collective action, and reflexive monitoring. Items were rated on a 5-point Likert scale (1=completely agree to 5=completely disagree, with 3=neutral). The NoMAD has high internal consistency in various health care settings and languages [46,47], including in mental health [48]."<br><br>Secondary outcome (uptake and effort) were developed specifically for this study and consisted of counting frequencies of "referral and completed treatments with adequate exposure levels" and "hours spent by implementers"		
**6a-ii) Describe whether and how "use" (including intensity of use/dosage) was defined/measured/monitored**		
Adequate exposure to the ItFits-toolkit was defined as: "the implementers completing modules 1, 2, and 3 within the exposure period (Figure 2)."		
**6a-iii) Describe whether, how, and when qualitative feedback from participants was obtained**		
An in-depth process evaluation (consisting of interviews, observations, and document analysis of implementers working with the ItFits-toolkit) was conducted alongside this implementation effectiveness study. This is currently under analysis and will be published as soon as possible.		
**6b) CONSORT: Any changes to trial outcomes after the trial commenced, with reasons**		
Detailed in methods section, including: "A total of 12 mental health service delivery organizations in 9 countries were included in the study (Table 1)."		
**7a) CONSORT: How sample size was determined**		
**7a-i) Describe whether and how expected attrition was taken into account when calculating the sample size**		
Sample size was calculated on basis of data simulations: "This study had a fixed number of 12 clusters by design. Mental health service delivery organizations participated based on their commitment to implementing iCBT services. For service providers, the sample size needed for sufficient power to test the use of the ItFits-toolkit on the degree of normalization (ie, NoMAD) was obtained from a power analysis using simulated data. As there was no prior knowledge concerning the NoMAD in detecting changes in normalization, we assumed a 5% increase in absolute normalization scores and an increase in the 3-month growth rate from 0.05 to 0.10. A cluster sample size of 15 service providers for each of the 12 mental health organizations per wave was estimated to be sufficient to achieve 80% power to detect the effect using a 2-sided test with a significance level α of .05. The first 2 data collection waves were used to obtain a stable sample, and recruitment was closed<br>in wave 3. Replacements were sought for those service providers who had dropped out of the study"		
**7b) CONSORT: When applicable, explanation of any interim analyses and stopping guidelines**		
Outcome measures were defined and did not change during the trail. They are described in detail in the protocol paper and in the current manuscript: "The primary outcome was the degree of normalization among service providers." [...] and " To complement the primary outcome, we assessed the effectiveness of the ItFits-toolkit using measures of uptake of<br>the iCBT service by patients (referral and completed treatments with adequate exposure levels) and implementation efficiency (operationalized by hours spent by implementers). These were used as secondary outcomes."		
**8a) CONSORT: Method used to generate the random allocation sequence**		
Randomisation followed a cluser-randomised stepped-wedge trial design: "Over a period of 30 months, the ItFits-toolkit was sequentially rolled out into 6 groups of 12 organizations (clusters). The clusters were randomly allocated with an interval of 3 months, at which the clusters crossed over from the control condition (IAU) to the experimental condition (ItFits-toolkit)."		
**8b) CONSORT: Type of randomisation; details of any restriction (such as blocking and block size)**		
NA. Trial sites were randomised following a stepped-wedge cluster design. Two sites were clustered in a group. 12 sites partook so we randomised 6 groups to different timepoints receiving the implementation toolkit.		
**9) CONSORT: Mechanism used to implement the random allocation sequence (such as sequentially numbered containers), describing any steps taken to conceal the sequence until interventions were assigned**		
Randomisation was performed by one person in the central trial coordination team. A month prior to cross-over the randomised implementation sites were informed to schedule the introduction training.		
**10) CONSORT: Who generated the random allocation sequence, who enrolled participants, and who assigned participants to interventions**		
Central trial coordination team consisting of 5 persons not involved in the implementation work and working with the toolkit, were involved in assignment and enrolment of implementers to the toolkit.		
**11a) CONSORT: Blinding - If done, who was blinded after assignment to interventions (for example, participants, care providers, those assessing outcomes) and how**		
**11a-i) Specify who was blinded, and who wasn't**		
Implementation-as-usual team that guided the control condition was blinded to the toolkit, allocation and subsequent analysis to not contaminate IAU condition.		
**11a-ii) Discuss e.g., whether participants knew which intervention was the "intervention of interest" and which one was the "comparator"**		
NA/ The design allowed for knowing conditions/ interventions		
**11b) CONSORT: If relevant, description of the similarity of interventions**		

NA; implementation study testing the effectiveness of an implementation toolkit.		
**12a) CONSORT: Statistical methods used to compare groups for primary and secondary outcomes**		
A detailed analysis plan was developed prior to opening the data: "Data for the primary outcome were analyzed using a 3-level linear mixed-effects modeling (LMM) approach [35] with normalization as the dependent variable and time (as a discrete variable) and intervention (ie, the ItFits-toolkit use) as independent variables. To account for expected intervention lag effect, a fractional term for the ItFits-toolkit use parameter was used to reflect the 6-month exposure time (0, 0.5, and 1). To account for a correlation structure in the outcome involving 3 nested levels, repeated measurements (L1) were clustered at the level of service providers (L2), and service providers were clustered at the organization level (L3). A temporal effect was assessed by testing the null hypothesis that the normalization level was constant over time when controlling for the effect of the ItFits-toolkit using ANOVA. A 2-sided test with a significance level α of .05 was used. Cohen d was used as a measure of the effect by which the modeled estimate was standardized by the pooled within-organization SD of the NoMAD scale at wave 1. Standard cutoff levels were applied (small effect: Cohen d≤0.2, medium effect: 0.2<Cohen d<0.8, and a large effect: Cohen d≥0.8)."		
**12a-i) Imputation techniques to deal with attrition / missing values**		
NA: "All observed data were included in the analyses following the intention-to-treat principle."		
**12b) CONSORT: Methods for additional analyses, such as subgroup analyses and adjusted analyses**		
Additional analysis were planned prior to opening the data: "Before analyzing and opening the data, various potentially confounding moderators were conceptually explored by the central research team following 2 workshops, using preliminary information from the qualitative process evaluation [33]. The role of staff in service delivery was selected as a potential moderator. Specifically, we assumed that staff who were more directly involved in iCBT service delivery such as psychologists and psychiatrists were likely to undergo a more extensive change process to normalize iCBT service delivery than those at a larger distance to the service delivery process such as referrers and administrators. For the secondary outcomes, service uptake (iCBT referral and completion) and effort (hours) were modeled following the same approach as for the primary outcome, except that a 2-level LMM was applied as these measures were collected at the organization level only (ie, not at the level of staff members but only waves [Level 1] clustered at the organization level [Level 2]). For exploratory purposes, measures of exposure to the ItFits-toolkit (module-based log as an indication of use), CSQ, SUS, and perceived impact and helpfulness of the ItFits-toolkit were assessed descriptively as an indication of usability, satisfaction, impact, and helpfulness of the ItFits-toolkit from the perspective of implementers. Perceived impact and helpfulness were measured using a VAS, with a continuous scale ranging from 1.0 10.0. The scale scores for SUS and CSQ were calculated using the respective prescribed scoring systems. For CSQ, summed item rating scores were used [56]. For SUS, the summed item ratings were converted to a 0 to 100 scale using a curved grading scale, with 68 points to be interpreted as neutral [58]."		
RESULTS		
**13a) CONSORT: For each group, the numbers of participants who were randomly assigned, received intended treatment, and were analysed for the primary outcome**		
Demographics are provided at the start of the results section in detail: "A total of 39 implementers in 12 mental health service delivery organizations used the ItFits-toolkit to implement the iCBT services" [...] And "A total of 456 iCBT service providers were included in this study (Table 3)." [...]		
**13b) CONSORT: For each group, losses and exclusions after randomisation, together with reasons**		
Only relevant for service deliverers (primary outcome): "The response rate of the service providers was high (78% across all 10 waves), resulting in 2884 complete data points. Approximately 30.9% (141/456) of the service providers were replaced because of study dropout during waves 3 to 10."		
**13b-i) Attrition diagram**		
NA, described in running text.		
**14a) CONSORT: Dates defining the periods of recruitment and follow-up**		
NA. Participants were replaced while running the trial. No periods were defined. However, the first three months (first two measurements) should be considered as a running-up period of the trail.		
**14a-i) Indicate if critical "secular events" fell into the study period**		
Various events occurred which is why this design was chosen. Events included natural hazards ranging from bushfires to earthquakes and of course COVID. Possible contamination and influence of these events are discussed: "number of unforeseen events, ranging from internal staff turnover to changing legislation and reimbursement models, and natural disasters, such as bush fires, earthquakes, and the COVID-19 pandemic, occurred as they did. All available data were used, and by applying a pragmatic stepped-wedge cluster randomized controlled trial study design with repeated measures and a psychometrically validated implementation outcome measure, the study allowed for these variations to provide an accurate representation of real-world implementation practice. By randomizing the moment of introduction of the ItFits-toolkit in the implementation of mental health organizations, both conditions had an equal chance of being exposed to the events that occurred during the trial."		
**14b) CONSORT: Why the trial ended or was stopped (early)**		
NA, trial was conducted and finished as planned.		
**15) CONSORT: A table showing baseline demographic and clinical characteristics for each group**		
Demographics of implementers was described (small group): "A total of 39 implementers in 12 mental health service delivery organizations used the ItFits-toolkit to implement the iCBT services. The group had a mean age of 42.6 (SD 10.1) years, and 69% (27/39) of the implementers were female. More than half (23/39, 59%) of the implementers had ≥6 years of work experience in mental health and were appointed as general project managers (13/39, 33%) or clinical researchers (12/39, 31%).".<br><br>Demographics of service deliverers (respondents to primary outcome) are provided in table 3.		
**15-i) Report demographics associated with digital divide issues**		
Not applicable to this implementation study (of an implementation toolkit).		
**16a) CONSORT: For each group, number of participants (denominator) included in each analysis and whether the analysis was by original assigned groups**		
**16-i) Report multiple "denominators" and provide definitions**		
Yes, for each analysis the number of participants is provided. More detailes including responses and model specifications are included in various annexes for the primary and secondary outcomes.		
**16-ii) Primary analysis should be intent-to-treat**		
All analyses were followed the intention-to-treat principle: "We relied on the capability of linear mixed-effects models to estimate model parameters in case of missing values under the Missing at Random assumption [59]."		
**17a) CONSORT: For each primary and secondary outcome, results for each group, and the estimated effect size and its precision (such as 95% confidence interval)**		
For all analyses the following principles were followed: "temporal effect was assessed by testing the null hypothesis that the normalization level was constant over time when controlling for the effect of the ItFits-toolkit using ANOVA. A 2-sided test with a significance level α of .05 was used. Cohen d was used as a measure of the effect by which the modeled estimate was standardized by the pooled within-organization SD of the NoMAD scale at wave 1. Standard cutoff levels were applied (small effect: Cohen d≤0.2, medium effect: 0.2<Cohen d<0.8, and a large effect: Cohen d≥0.8)."		
**17a-i) Presentation of process outcomes such as metrics of use and intensity of use**		
All implementers (itfits-toolkit users) received the adequate exposure as defined. Detailed use is subject of the proces evaluation which is not subject of this study and will be published separately.		
**17b) CONSORT: For binary outcomes, presentation of both absolute and relative effect sizes is recommended**		
NA, no binary outcomes were used.		
**18) CONSORT: Results of any other analyses performed, including subgroup analyses and adjusted analyses, distinguishing pre-specified from exploratory**		
All secondary and exploratory analyses are reported as planned. These include changes in uptake and effort (secondary outcomes) and perceived usability and acceptability of the toolkit by implementers (exploratory outcomes).		
**18-i) Subgroup analysis of comparing only users**		
For the primary outcome, a subgroup analysis was conducted as planned comparing service delivers more close to the delivery process (e.g. therapists) with those further away (e.g. administrators). The analysis was planned prior to opening the data.		
**19) CONSORT: All important harms or unintended effects in each group**		
NA, this is an implementation study and we did not find any harms or unintended effects that can be attributed to conducting this study or using the implementation toolkit.		
**19-i) Include privacy breaches, technical problems**		
No data or privacy breaches occurred. Toolkit and data collection system functioned as required and protocolled.		
**19-ii) Include qualitative feedback from participants or observations from staff/researchers**		
This is subject of an in-depth process evaluation and will be reported in a separate paper detailing how implementers used the toolkit.		
DISCUSSION		
**20) CONSORT: Trial limitations, addressing sources of potential bias, imprecision, multiplicity of analyses**		
**20-i) Typical limitations in ehealth trials**		

"Some methodological limitations should be considered when interpreting the results. One aspect was that IAU activities cannot be undone once embarked upon. The results might be influenced by carryover effects and intervention lag effects within the service delivery organizations." [...] "A second methodological limitation relates to outcome normalization, as measured by the NoMAD. This questionnaire was developed with precision and methodological rigor [45,72] and has been psychometrically validated in various studies in various settings [46-48]. However, these studies have used cross-sectional samples, and the psychometric sensitivity of NoMAD to longitudinal change is yet to be explored." [...] "Another factor that might have led to an underestimation of the effect was that some implementers had a background in research or were practicing research and that some had prior experience in implementing iCBT services. This experience might, for example, have affected adherence to some principles of the toolkit such as “being different” in implementing the iCBT service. Similarly, participating in a large-scale international research project designed to address implementation issues might have influenced implementers in their knowledge of and setting priorities in their implementation work."		
**21) CONSORT: Generalisability (external validity, applicability) of the trial findings**		
**21-i) Generalizability to other populations**		
"A strength of this trial was its high ecological validity. We managed to study a diverse group of implementers and iCBT service providers that were representative of routine care in 12 mental health service organizations in 8 European countries and Australia. Representing routine care mental health service delivery practice, the way in which mental health services were operationalized and delivered, including the clinical focus, guidance modalities, technical platform, and availability of mental health professionals and their experience with cognitive behavioral therapy, varied among the mental health service delivery organizations."		
**21-ii) Discuss if there were elements in the RCT that would be different in a routine application setting**		
We followed routine implementation practice as much as possible and did not, as far as we know, infringe routine care settings. The premise was to conduct an implementation study under routine conditions.		
**22) CONSORT: Interpretation consistent with results, balancing benefits and harms, and considering other relevant evidence**		
**22-i) Restate study questions and summarize the answers suggested by the data, starting with primary outcomes and process outcomes (use)**		
"This study sought to examine whether the ItFits-toolkit leads to better implementation outcomes than IAU in implementing iCBT services. In comparison with IAU, the ItFits-toolkit has a small statistically significant effect on normalization levels in iCBT service providers. The toolkit did not have an effect on iCBT service uptake by patients, and the implementers did not spend more time using the toolkit. ItFits-toolkit users regarded the toolkit generally as usable and were satisfied with it. These findings fit the general pattern across tailoring studies [27]"		
**22-ii) Highlight unanswered new questions, suggest future research**		
The discussion raised several new research questions. "First, the effect was small. Depending on the research question and context, a small effect can be of importance to informing implementation processes. How this effect size should be interpreted in terms of practical improvement of implementation outcomes is yet to be determined." And "Second, to optimize the effectiveness of the ItFits-toolkit, a dismantling study can be used to determine which components of self-guided tailored implementation contributed most to the outcomes of the ItFits-toolkit", and: "Third, implementation work is dynamic, takes time, and is context specific. The outcome measures used in this study showed little variation over time. Moreover, the normalization levels found in this study declined over time. This requires further discussion in theory development (NPT) and verification of the NoMAD with other instruments measuring implementation outcomes longitudinally and their sensitivity to change over time."		
Other information		
**23) CONSORT: Registration number and name of trial registry**		
"The study protocol was published [31], and the study was registered with ClinicalTrials.gov (NCT03652883)."		
**24) CONSORT: Where the full trial protocol can be accessed, if available**		
Bührmann L, Schuurmans J, Ruwaard J, Fleuren M, Etzelmüller A, Piera-Jiménez J, Finch T, Rapley T, Potthoff S, Aouizerate B, Batterham P, Calear A, Christensen H, Duedal Pederson C, Ebert D, van der Eycken E, Fanaj N, van Genugten C, Hanssen D, Hegerl U, Hug J, Kleiboer A, Mathiasen K, May C, Mustafa S, Oehler C, Cerga Pashoja A, Pope C, Qirjako G, Rosmalen, J, Sacco Y, Samalin L, Skjøth M, Tarp K, Titzler I, Zanalda E, Zbukvic I, Smit J, Riper H, Vis C, (2020). Tailored implementation of internet-based cognitive behavioural therapy in the multinational context of the ImpleMentAll project: a study protocol for a stepped wedge cluster randomized trial. Trials 21, 893		
**25) CONSORT: Sources of funding and other support (such as supply of drugs), role of funders**		
"This study was funded by the European Union’s Horizon 2020 research and innovation program under grant agreement 733025 and by the National Health and Medical Research Council EU-program by the Australian Government (grant 1142363). The funding bodies had no influence on the design of the study."		
**X26-i) Comment on ethics committee approval**		
NB: this is an implementation study and not a medical intervention study. "Each local research team translated and adapted the generic study protocol to the local requirements and submitted it for review to competent local medical ethical review committees. All committees either approved the study or provided a confirmation letter that it was exempt from approval as the study was not considered as medical research. A portfolio of the ethical documentation can be accessed through Deliverable 23 (D9.1, H – Requirement No. 1), which was submitted to the European Commission (Horizon 2020, Grant Agreement 733025). The study participants signed an informed consent form indicating the purpose of the study and the nature, use, and management of their data. The study protocol was published [31], and the study was registered with ClinicalTrials.gov (NCT03652883)."		
**x26-ii) Outline informed consent procedures**		
Part of the ethical approvals: "The study participants signed an informed consent form indicating the purpose of the study and the nature, use, and management of their data."		
**X26-iii) Safety and security procedures**		
NA; local clinical guidelines were followed. Note, it concerned the implementation of existing evidence-based iCBT interventions.		
**X27-i) State the relation of the study team towards the system being evaluated**		
NA not included in the manuscript		
